# Supplementary material for: Shoulder specific exercise therapy is effective in reducing chronic shoulder pain: A network meta-analysis
Source: PLoS One. 2024 Apr 29;19(4):e0294014. doi: 10.1371/journal.pone.0294014 (PMC11057978; doi:10.1371/journal.pone.0294014)
Supplement: S2 Appendix — (DOCX) [file pone.0294014.s002.docx]

**SEARCH STRATEGIES**

# Database: MEDLINE Ovid MEDLINE(R) and Epub Ahead of Print, In-Process &amp; Other NonIndexed Citations and Daily; 1946 to May 22, 2022

1. Bursitis/ and (gleno* or rotator cuff* or shoulder*).mp. (1493)
2. Rotator Cuff/ (7642)
3. Rotator Cuff Injuries/ (7019)
4. exp Scapula/ (8718)
5. Shoulder/ (14725)
6. Shoulder Impingement Syndrome/ (1901)
7. Shoulder Injuries/ (4946)
8. Shoulder Joint/ (20642)
9. Shoulder Pain/ (5528)
10. Tendinopathy/ and (gleno* or rotator cuff* or shoulder*).mp. (1614)
11. Tenosynovitis/ and (gleno* or rotator cuff* or shoulder*).mp. (177)
12. ((acromion* or coracoid* or gleno* or infra-spinatus* or infraspinatus* or rotator cuff* or shoulder* or scapul* or supra-spinatus* or supraspinatus* or teres minor*) adj3 (bursiti* or capsuliti* or degenerati* or disease* or disorder* or imping* or inflam* or injur* or pain* or patholog* or problem* or ruptur* or stiff* or strain* or stress* or tear* or tendin* or tendoni* or tendono* or tenosyno* or torn* or trauma* or weak* or wear*)).tw,kf.

(28923)

1. frozen shoulder*.tw,kf. (1280)
2. SLAP tear*.tw,kf. (254)
3. or/1-14 [Combined MeSH & text words for shoulder pathology] (59861)
4. Acupressure/ (898)
5. Acupuncture Therapy/ (18740)
6. Acupuncture/ (1932)
7. Adrenal Cortex Hormones/tu [Therapeutic use] (34233)
8. Anti-Inflammatory Agents/ (89508)
9. Anti-Inflammatory Agents, Non-Steroidal/ (71022)
10. Conservative Treatment/ (4586)
11. Cryotherapy/ (5585)
12. Electric Stimulation Therapy/ (21638)
13. exp Exercise Therapy/ (59571)
14. General Practitioners/ (9648)
15. Glucocorticoids/ (68999)
16. exp Hyperthermia, Induced/ (36233)
17. Injections, Intra-Articular/ (8850)
18. Methylprednisolone/ (19872)
19. "Physical and Rehabilitation Medicine"/ (3494)
20. Physical Therapy Modalities/ (39492)
21. Physicians, Family/ (16949)
22. Physicians, Primary Care/ (4183)
23. Physicians' Offices/ (1787)
24. *Postural Balance/ (16599)
25. *Posture/ (29281)
26. Primary Care/ (87908)
27. Primary Health Care/ (87908)
28. Rehabilitation/ (18654)
29. Ultrasonography, Interventional/ (26449)
30. Watchful Waiting/ (4903)
31. (acupunctur* or acupressure*).tw,kf. (26396)
32. ((adjust* or intervention* or modif* or stabil*) adj2 postur*).tw,kf. (6061)
33. (anti-inflammator* or antiinflammator* or NSAID*).tw,kf. (218606)
34. ((appl* or pack* or pad*) adj2 (hot or heat*)).tw,kf. (3943)
35. ((appl* or pack or pad*) adj2 (ice or cold)).tw,kf. (2065)
36. ((care* or healthcare*) adj2 primary).tw,kf. (172455)
37. chiropract*.tw,kf. (6495)
38. (cold therap* or cryotherap*).tw,kf. (8541)
39. ((conservative* or non-operative* or non-surgical* or nonoperative* or nonsurgical*) adj3 (manage* or therap* or treat*)).tw,kf. (116721)
40. ((cortico* or cortiso* or glucocortico* or prednison* or steroid*) adj2 (inject* or shot*)).tw,kf. (116721)
41. ((doctor* or physician*) adj office*).tw,kf. (5269)
42. dry needling*.tw,kf. (779)
43. (exercis* adj2 therap*).tw,kf. (9939)
44. general practi*.tw,kf. (89189)
45. icing.tw,kf. (949)
46. interferential current*.tw,kf. (224)
47. manual therap*.tw,kf. (3189)
48. motion therap*.tw,kf. (107)
49. (physio-therap* or physiotherap* or physical therap*).tw,kf. (58434)
50. rehab*.tw,kf. (204126)
51. ((resistance or strength* or weight*) adj1 train*).tw,kf. (17398)
52. (stretches or stretching).tw,kf. (39391)
53. (wait* adj2 watch*).tw,kf. (4477)
54. or/16-65 [Combined MeSH & text words for conservative management] (1314570)
55. and/15,66 [Combined concepts for shoulder pathology & conservative management] (12609)
56. randomized controlled trial.pt. (568162)
57. controlled clinical trial.pt. (94879)
58. randomized.ab. (560853)
59. placebo.ab. (228198)
60. clinical trials as topic.sh. (199869)
61. randomly.ab. (381983)
62. trial.ti. (262251)
63. or/68-74 (1445842)
64. exp Animals/ not Humans/ (5004231)
65. 75 not 76 [Cochrane Highly Sensitive Search Strategy for identifying randomized trials in MEDLINE: sensitivity- and precision-maximizing version (2008 revision); Lefebvre C, et al. Retrieved: http://handbook.cochrane.org/chapter_6/6_searching_for_studies.htm] (1329754)
66. and/67,77 [RCT filter applied] (2425)
67. clinical trial.pt. (535034)
68. comparative study.pt. (1911325)
69. exp case control studies/ (1320425)
70. exp cohort studies/ (2347670)
71. (cohort adj (study or studies)).tw. (271563)
72. (comparative adj (study or studies)).tw. (113407)
73. control.tw. (2861354)
74. controlled.tw. (885936)
75. ((design* or studies or study or test*) adj2 (post* adj2 pre*)).tw. (21526)
76. (follow up adj (study or studies)).tw. (53519)
77. longitudinal.tw. (291178)
78. (non-random* or nonrandom*).tw. (44552)
79. (observational adj (study or studies)).tw. (139420)
80. (quasi-random* or quasirandom*).tw. (5350)
81. retrospective.tw. (659754)
82. or/79-93 (7630528)
83. exp Animals/ not Humans/ (5004231)
84. (animal* or bovine* or calves or camel* or canine* or cat or cats or chimp* or dog or dogs or equine* or feline* or goat* or hamster* or horse* or llama* or mice* or monkey* or mouse* or pig or piglet* or pigs or porcine* or primate* or rabbit* or rat or rats or rodent* or sheep* or simian* or swine*).ti. (2403377)
85. 94 not (95 or 96) [Observational study filter - not validated] (6359238)
86. and/67,97 [Observational filter applied] (5665)
87. or/78,98 [Shoulder pathologies & conservative management w/ combined study design filters] (6006)
88. exp Child/ not (exp Adult/ and exp Child/) (1329811)
89. (adolescen* or child* or infan* or juvenile* or neonat* or p?ediatric* or youth*).ti. (1523889)
90. 99 not (100 or 101) [Exclude pediatric studies] (5869)
91. exp Aged/ not ((Adult/ or Middle Aged/ or Young Adult/) and exp Aged/) (738577)
92. (aged or elder* or geriatic* or older adult* or senior*).ti. (248761)
93. 102 not (103 or 104) [Exclude older adult studies] (5697)
94. *Anesthetics, Local/ (23822)
95. *Brachial Plexus Block/ (667)
96. (an?esth* or brachial plexus block*).ti. (156682)
97. 105 not (106 or 107 or 108) [Exclude anesthetic interventions] (5481)
98. exp *Arthritis/ (237401)
99. (arthriti* or osteoarthri* or OA).ti. (148522)
100. 109 not (110 or 111) [Exclude arthritis studies] (5262)
101. exp *Breast Neoplasms/ (279440)
102. exp *"Head and Neck Neoplasms"/ (293155)
103. cancer*.ti. (1165335)
104. 112 not (113 or 114 or 115) [Exclude cancer studies] (5118)
105. exp *Perioperative Care/ (58494)
106. (intraop* or operative* or periop* or postop*).ti. (188666)
107. 116 not (117 or 118) [Exclude perioperative interventions] (4938)
108. *Shoulder Dislocation/ (5360)
109. (bankart* or dislocat* or instab* or subluxat*).ti. (57816)
110. 119 not (120 or 121) [Exclude dislocation studies] (4574)
111. exp *Stroke/ (122747)
112. (isch?em* or stroke* or TIA).ti. (260051)
113. 122 not (123 or 124) [Exclude stroke studies] (4359)
114. (editorial or comment or letter or newspaper article).pt. (2077106)
115. 125 not 126 [Exclude opinion pieces] (4306)
116. limit 127 to english (4041)
117. remove duplicates from 128 (4031)

**Database: Wiley Cochrane Trials (CENTRAL)**

**Strategy:**

| ID Search  #1 [mh ^"Bursitis"] AND (gleno* or (rotator NEAR/1 cuff*) or  shoulder*):ti,ab,kw   309  #2 [mh ^"Rotator Cuff"]  436  #3 [mh ^"Rotator Cuff Injuries"]   597  #4 [mh "Scapula"] 208  #5 [mh ^"Shoulder"] 642  #6 [mh ^"Shoulder Impingement Syndrome"] 415  #7 [mh ^"Shoulder Injuries"] 119  #8 [mh ^"Shoulder Joint"] 816  #9 [mh ^"Shoulder Pain"]  1093  #10 [mh ^"Tendinopathy"] and (gleno* or (rotator NEAR/1 cuff*) or  shoulder*):ti,ab,kw 251  #11 [mh ^"Tenosynovitis"] and (gleno* or (rotator NEAR/1 cuff*) or  shoulder*):ti,ab,kw 15  #12 ((acromion* or coracoid* or gleno* or (infra NEAR/1 spinatus*) or infraspinatus* or (rotator NEAR/1 cuff*) or shoulder* or scapul* or (supra NEAR/1 spinatus*) or supraspinatus* or (teres NEAR/1 minor*)) NEAR/3 (bursiti* or capsuliti* or degenerati* or disease* or disorder* or imping* or inflam* or injur* or pain* or patholog* or problem* or ruptur* or stiff* or strain* or stress* or tear* or tendin* or tendoni* or tendono* or tenosyno* or torn* or trauma* or weak* or wear*)):ti,ab,kw 6660  #13 (frozen NEAR/1 shoulder*):ti,ab,kw   507  #14 (SLAP NEAR/1 tear*):ti,ab,kw   5  #15 #1 OR #2 OR #3 OR #4 OR #5 OR #6 OR #7 OR #8 OR #9 OR #10 OR #11 OR #12 OR #13 OR #14  7603  #16 [mh ^"Acupressure"]  419  #17 [mh ^"Acupuncture Therapy"]  3315  #18 [mh ^"Acupuncture"]  162  #19 [mh ^"Adrenal Cortex Hormones"/tu]  1394  #20 [mh ^"Anti-Inflammatory Agents"]  6162  #21 [mh ^"Anti-Inflammatory Agents, Non-Steroidal"] 6766  #22 [mh ^"Conservative Treatment"]  178  #23 [mh ^"Cryotherapy"] 763  #24 [mh ^"Electric Stimulation Therapy"] 2026  #25 [mh "Exercise Therapy"] 16029  #26 [mh ^"General Practitioners"]  332  #27 [mh ^"Glucocorticoids"] 4777  #28 [mh "Hyperthermia, Induced"] 1807  #29 [mh ^"Injections, Intra-Articular"] 1423  #30 [mh ^"Methylprednisolone"] 2820  #31 [mh ^"Physical and Rehabilitation Medicine"] 20  #32 [mh ^"Physical Therapy Modalities"]  4092  #33 [mh ^"Physicians, Family"] 462  #34 [mh ^"Physicians, Primary Care"] 175  #35 [mh ^"Postural Balance"] 3139  #36 [mh ^"Posture"]  3485  #37 [mh ^"Primary Health Care"] 4685  #38 [mh ^"Rehabilitation"]  322  #39 [mh ^"Ultrasonography, Interventional"]  2249  #40 [mh ^"Watchful Waiting"] 365  #41 (acupunctur* or acupressure*):ti,ab,kw 17527  #42 ((adjust* or intervention* or modif* or stabil*) NEAR/2 postur*):ti,ab,kw 1443  #43 ((anti NEAR/1 inflammator*) or antiinflammator* or NSAID*):ti,ab,kw  34729  #44 ((appl* or pack* or pad*) NEAR/2 (hot or heat*)):ti,ab,kw 934  #45 ((appl* or pack or pad*) NEAR/2 (ice or cold)):ti,ab,kw  776  #46 chiropract*:ti,ab,kw 1190  #47 ((cold NEAR/1 therap*) or cryotherap*):ti,ab,kw 2556  #48 ((conservative* or (non NEAR/1 operative*) or (non NEAR/1 surgical*) or nonoperative* or nonsurgical*) NEAR/3 (manage* or therap* or treat*)):ti,ab,kw 12148  #49 ((cortico* or cortiso* or glucocortico* or prednison* or steroid*) NEAR/2 (inject* or shot*)):ti,ab,kw 3421  #50 ((doctor* or physician*) NEAR/1 office*):ti,ab,kw 627  #51 (dry NEAR/1 needling*):ti,ab,kw  805  #52 (exercis* NEAR/2 therap*):ti,ab,kw 18190  #53 (general NEAR/1 practi*):ti,ab,kw 12388  #54 icing:ti,ab,kw 57  #55 (interferential NEAR/1 current*):ti,ab,kw 267  #56 (manual NEAR/1 therap*):ti,ab,kw 2062  #57 (motion NEAR/1 therap*):ti,ab,kw  221  #58 ((physio NEAR/1 therap*) or physiotherap* or (physical NEAR/1 therap*)):ti,ab,kw 26484  #59 rehab*:ti,ab,kw 56888  #60 ((resistance or strength* or weight*) NEAR/1 train*):ti,ab,kw 13510  #61 (stretches or stretching):ti,ab,kw 6573  #62 (wait* NEAR/2 watch*):ti,ab,kw 1101  #63 #16 OR #17 OR #18 OR #19 OR #20 OR #21 OR #22 OR #23 OR #24 OR #25 OR #26 OR #27 OR #28 OR #29 OR #30 OR #31 OR #32 OR #33 OR #34 OR #35 OR #36 OR #37 OR #38 OR #39 OR #40 OR #41 OR #42 OR #43 OR #44 OR #45 OR #46 OR #47 OR #48 OR #49 OR #50 OR #51 OR #52 OR #53 OR #54 OR #55 OR #56 OR #57 OR #58 OR #59 OR #60 OR #61 OR #62 192838  #64 #63 AND #15  3968  #65 [mh "Child"] NOT ([mh "Adult"] AND [mh "Child"])  43935  #66 (adolescen* or child* or infan* or juvenile* or neonat* or paediatric* or pediatric* or youth*):ti 138440  #67 #64 NOT (#65 OR #66) 3944  #68 [mh "Aged"] NOT (([mh ^"Adult"] OR [mh ^"Middle Aged"] OR [mh ^"Young Adult"]) AND [mh "Aged"]) 26097  #69 (aged or elder* or geriatic* or older adult* or senior*):ti 36989  #70 #67 NOT (#68 OR #69) 3892  #71 [mh ^"Anesthetics, Local"] 9079  #72 [mh ^"Brachial Plexus Block"] 212  #73 (anaesth* or anesth* or ("brachial plexus" NEAR/1 block*)):ti 30328  #74 #70 NOT (#71 OR #72 OR #73) 3678  #75 [mh "Arthritis"] 17202  #76 (arthriti* or osteoarthri* or OA):ti 26564  #77 #74 NOT (#75 OR #76) 3561  #78 [mh "Breast Neoplasms"] 14489  #79 [mh "Head and Neck Neoplasms"] 6517  #80 cancer*:ti  110905  #81 #77 NOT (#78 OR #79 OR #80) 3421  #82 [mh "Perioperative Care"]  12780  #83 (intraop* or operative* or periop* or postop*):ti 42731  #84 #81 NOT (#82 OR #83) 3286  #85 [mh ^"Shoulder Dislocation"] 165  #86 (bankart* or dislocat* or instab* or subluxat*):ti 1956  #87 #84 NOT (#85 OR #86) 3186  #88 [mh "Stroke"]  11365  #89 (ischaem* or ischem* or stroke* or TIA):ti 42334  #90 #87 NOT (#88 OR #89) 2982 |
| --- |

**Database: CINAHL Plus with Full Text via EBSCOhost**

**Strategy:**

| \| **#** \| **Query** \| **Limiters/Expanders** \| **Results** \| \| --- \| --- \| --- \| --- \| \| S1 \| (MH "Bursitis") and (TI (gleno* or "rotator cuff*" or shoulder*) or AB (gleno* or "rotator cuff*" or shoulder*)) \| Search modes - Find all my search terms \| 321 \| \| S2 \| (MH "Rotator Cuff+") \| Search modes - Find all my search terms \| 3,589 \| \| S3 \| (MH "Rotator Cuff Injuries") \| Search modes - Find all my search terms \| 3,177 \| \| S4 \| (MH "Scapula+") \| Search modes - Find all my search terms \| 3,021 \| \| S5 \| (MH "Shoulder") \| Search modes - Find all my search terms \| 7,385 \| \| S6 \| (MH "Shoulder Impingement Syndrome") \| Search modes - Find all my search terms \| 1,476 \| \| S7 \| (MH "Shoulder Injuries") \| Search modes - Find all my search terms \| 2,312 \| \| S8 \| (MH "Shoulder Joint+") \| Search modes - Find all my search terms \| 6,845 \| \| S9 \| (MH "Shoulder Pain") \| Search modes - Find all my search terms \| 4,463 \| \| S10 \| (MH "Tendinopathy") and (TI (gleno* or "rotator cuff*" or shoulder*) or AB (gleno* or "rotator cuff*" or shoulder*)) \| Search modes - Find all my search terms \| 630 \| \| S11 \| (MH "Tenosynovitis+") and (TI (gleno* or "rotator cuff*" or shoulder*) or AB (gleno* or "rotator cuff*" or shoulder*)) \| Search modes - Find all my search terms \| 62 \| \| S12 \| TI ((acromion* or coracoid* or gleno* or "infra spinatus*" or infraspinatus* or "rotator cuff*" or shoulder* or scapul* or "supra spinatus*" or supraspinatus* or "teres minor*") N3 (bursiti* or capsuliti* or degenerati* or disease* or disorder* or imping* or inflam* or injur* or pain* or patholog* or problem* or ruptur* or stiff* or strain* or stress* or tear* or tendin* or tendoni* or tendono* or tenosyno* or torn* or trauma* or weak* or wear*)) or AB ((acromion* or coracoid* or gleno* or "infra spinatus*" or infraspinatus* or "rotator cuff*" or shoulder* or scapul* or "supra spinatus*" or supraspinatus* or "teres minor*") N3 (bursiti* or capsuliti* or degenerati* or disease* or disorder* or imping* or inflam* or injur* or pain* or patholog* or problem* or ruptur* or stiff* or strain* or stress* or tear* or tendin* or tendoni* or tendono* or tenosyno* or torn* or trauma* or weak* or wear*)) \| Search modes - Find all my search terms \| 14,172 \| \| S13 \| TI "frozen shoulder*" or AB "frozen shoulder*" \| Search modes - Find all my search terms \| 561 \| \| S14 \| TI "SLAP tear*" or AB "SLAP tear*" \| Search modes - Find all my search terms \| 136 \| \| S15 \| S1 OR S2 OR S3 OR S4 OR S5 OR S6 OR S7 OR S8 OR S9 OR S10 OR S11 OR S12 OR S13 OR S14 \| Search modes - Find all my search terms \| 27,893 \| \| S16 \| (MH "Acupressure") \| Search modes - Find all my search terms \| 1,500 \| \| S17 \| (MH "Acupuncture") \| Search modes - Find all my search terms \| 15,722 \| \| S18 \| (MH "Adrenal Cortex Hormones/TU") \| Search modes - Find all my search terms \| 8,725 \| \| S19 \| (MH "Antiinflammatory Agents") \| Search modes - Find all my search terms \| 13,061 \| \| S20 \| (MH "Antiinflammatory Agents, Non-Steroidal") \| Search modes - Find all my search terms \| 15,032 \| \| S21 \| (MM "Balance, Postural") \| Search modes - Find all my search terms \| 11,615 \| \| S22 \| (MH "Cryotherapy") \| Search modes - Find all my search terms \| 3,145 \| \| S23 \| (MH "Dry Needling") \| Search modes - Find all my search terms \| 402 \| \| S24 \| (MH "Glucocorticoids+") \| Search modes - Find all my search terms \| 19,618 \| \| S25 \| (MH "Heat-Cold Application") \| Search modes - Find all my search terms \| 848 \| \| S26 \| (MH "Hyperthermia, Induced") \| Search modes - Find all my search terms \| 1,387 \| \| S27 \| (MH "Injections, Intraarticular") \| Search modes - Find all my search terms \| 3,144 \| \| S28 \| (MH "Methylprednisolone") \| Search modes - Find all my search terms \| 3,440 \| \| S29 \| (MH "Physical Medicine") \| Search modes - Find all my search terms \| 1,941 \| \| S30 \| (MH "Physical Therapy") \| Search modes - Find all my search terms \| 37,704 \| \| S31 \| (MH "Physicians, Family") \| Search modes - Find all my search terms \| 22,237 \| \| S32 \| (MM "Posture") \| Search modes - Find all my search terms \| 7,888 \| \| S33 \| (MH "Primary Health Care") \| Search modes - Find all my search terms \| 70,750 \| \| S34 \| (MH "Rehabilitation") \| Search modes - Find all my search terms \| 19,549 \| \| S35 \| (MH "Therapeutic Exercise+") \| Search modes - Find all my search terms \| 60,172 \| \| S36 \| TI (acupunctur* or acupressure*) or AB (acupunctur* or acupressure*) \| Search modes - Find all my search terms \| 16,327 \| \| S37 \| TI ((adjust* or intervention* or modif* or stabil*) N2 postur*) or AB ((adjust* or intervention* or modif* or stabil*) N2 postur*) \| Search modes - Find all my search terms \| 2,969 \| \| S38 \| TI ("anti inflammator*" or antiinflammator* or NSAID*) or AB ("anti inflammator*" or antiinflammator* or NSAID*) \| Search modes - Find all my search terms \| 35,327 \| \| S39 \| TI ((appl* or pack* or pad*) N2 (hot or heat*)) or AB ((appl* or pack* or pad*) N2 (hot or heat*)) \| Search modes - Find all my search terms \| 985 \| \| S40 \| TI ((appl* or pack or pad*) N2 (ice or cold)) or AB ((appl* or pack or pad*) N2 (ice or cold)) \| Search modes - Find all my search terms \| 937 \| \| S41 \| TI ((care* or healthcare*) N2 primary) or AB ((care* or healthcare*) N2 primary \| Search modes - Find all my search terms \| 103,162 \| \| S42 \| TI chiropract* or AB chiropract* \| Search modes - Find all my search terms \| 15,962 \| \| S43 \| TI ("cold therap*" or cryotherap*) or AB ("cold therap*" or cryotherap*) \| Search modes - Find all my search terms \| 2,059 \| \| S44 \| TI ((conservative* or "non operative*" or "non surgical*" or nonoperative* or nonsurgical*) N3 (manage* or therap* or treat*)) or AB ((conservative* or "non operative*" or "non surgical*" or nonoperative* or nonsurgical*) N3 (manage* or therap* or treat*)) \| Search modes - Find all my search terms \| 28,699 \| \| S45 \| TI ((cortico* or cortiso* or glucocortico* or prednison* or steroid*) N2 (inject* or shot*)) or AB ((cortico* or cortiso* or glucocortico* or prednison* or steroid*) N2 (inject* or shot*)) \| Search modes - Find all my search terms \| 4,504 \| \| S46 \| TI ((doctor* or physician*) N1 office*) or AB ((doctor* or physician*) N1 office*) \| Search modes - Find all my search terms \| 12,635 \| \| S47 \| TI "dry needling*" or AB "dry needling*" \| Search modes - Find all my search terms \| 747 \| \| S48 \| TI (exercis* N2 therap*) or AB (exercis* N2 therap*) \| Search modes - Find all my search terms \| 5,524 \| \| S49 \| TI "general practi*" or AB "general practi*" \| Search modes - Find all my search terms \| 33,898 \| \| S50 \| TI icing or AB icing \| Search modes - Find all my search terms \| 137 \| \| S51 \| TI "interferential current*" or AB "interferential current*" \| Search modes - Find all my search terms \| 157 \| \| S52 \| TI "manual therap*" or AB "manual therap*" \| Search modes - Find all my search terms \| 2,920 \| \| S53 \| TI "motion therap*" or AB "motion therap*" \| Search modes - Find all my search terms \| 32 \| \| S54 \| TI ("physio therap*" or physiotherap* or "physical therap*") or AB ("physio therap*" or physiotherap* or "physical therap*") \| Search modes - Find all my search terms \| 46,977 \| \| S55 \| TI rehab* or AB rehab* \| Search modes - Find all my search terms \| 108,495 \| \| S56 \| TI ((resistance or strength* or weight*) N1 train*) or AB (resistance or strength* or weight*) N1 train*) \| Search modes - Find all my search terms \| 10,507 \| \| S57 \| TI (stretches or stretching) or AB (stretches or stretching) \| Search modes - Find all my search terms \| 9,641 \| \| S58 \| TI (wait* N2 watch*) or AB (wait* N2 watch*) \| Search modes - Find all my search terms \| 1,371 \| \| S59 \| S16 OR S17 OR S18 OR S19 OR S20 OR S21 OR S22 OR S23 OR S24 OR S25 OR S26 OR S27 OR S28 OR S29 OR S30 OR S31 OR S32 OR S33 OR S34 OR S35 OR S36 OR S37 OR S38 OR S39 OR S40 OR S41 OR S42 OR S43 OR S44 OR S45 OR S46 OR S47 OR S48 OR S49 OR S50 OR S51 OR S52 OR S53 OR S54 OR S55 OR S56 OR S57 OR S58 \| Search modes - Find all my search terms \| 547,705 \| \| S60 \| S15 AND S59 \| Search modes - Find all my search terms \| 8,326 \| \| S61 \| (MH "Clinical Trials+") \| Search modes - Find all my search terms \| 337,511 \| \| S62 \| (MH "Placebos") \| Search modes - Find all my search terms \| 13,676 \| \| S63 \| (MH "Quantitative Studies") \| Search modes - Find all my search terms \| 31,875 \| \| S64 \| (MH "Random Assignment") \| Search modes - Find all my search terms \| 73,608 \| \| S65 \| PT Clinical trial \| Search modes - Find all my search terms \| 112,441 \| \| S66 \| TX (allocat* N1 random*) \| Search modes - Find all my search terms \| 18,709 \| \| S67 \| TX ( (doubl* N1 blind*) or (doubl* N1 mask*) ) \| Search modes - Find all my search terms \| 1,251,203 \| \| S68 \| TX clinic* N1 trial* \| Search modes - Find all my search terms \| 393,497 \| \| S69 \| TX placebo* \| Search modes - Find all my search terms \| 112,366 \| \| S70 \| TX randomi* control* trial* \| Search modes - Find all my search terms \| 369,395 \| \| S71 \| TX ( (singl* N1 blind*) or (singl* N1 mask*) ) \| Search modes - Find all my search terms \| 23,248 \| \| S72 \| TX ( (trebl* N1 blind*) or (trebl* N1 mask*) ) \| Search modes - Find all my search terms \| 9 \| \| S73 \| TX ( (tripl* N1 blind*) or (tripl* N1 mask*) ) \| Search modes - Find all my search terms \| 984 \| \| S74 \| S61 OR S62 OR S63 OR S64 OR S65 OR S66 OR S67 OR S68 OR S69 OR S70 OR S71 OR S72 OR S73 \| Search modes - Find all my search terms \| 1,767,159 \| \| S75 \| S60 AND S74 \| Search modes - Find all my search terms \| 3,010 \| \| S76 \| (MH "Control Group") \| Search modes - Find all my search terms \| 12,927 \| \| S77 \| (MH "Correlational Studies") \| Search modes - Find all my search terms \| 28,630 \| \| S78 \| (MH "Matched-Pair Analysis") \| Search modes - Find all my search terms \| 2,130 \| \| S79 \| (MH "Nonexperimental Studies+") \| Search modes - Find all my search terms \| 858,711 \| \| S80 \| TI "case control*" or AB "case control*" \| Search modes - Find all my search terms \| 41,065 \| \| S81 \| TI cohort* or AB cohort* \| Search modes - Find all my search terms \| 269,197 \| \| S82 \| TI "comparative stud*" or AB "comparative stud*" \| Search modes - Find all my search terms \| 17,361 \| \| S83 \| TI (control or controlled) or AB (control or controlled) \| Search modes - Find all my search terms \| 730,468 \| \| S84 \| TI "cross sectional" or AB "cross sectional" \| Search modes - Find all my search terms \| 186,557 \| \| S85 \| TI "descriptive stud*" or AB "descriptive stud*" \| Search modes - Find all my search terms \| 22,232 \| \| S86 \| TI ((design* or studies or study or test*) N2 (post* or pre*)) or AB ((design* or studies or study or test*) N2 (post* or pre*)) \| Search modes - Find all my search terms \| 379,109 \| \| S87 \| TI ("follow up" or followup) or AB ("follow up" or followup) \| Search modes - Find all my search terms \| 301,167 \| \| S88 \| TI observational or AB observational \| Search modes - Find all my search terms \| 87,026 \| \| S89 \| TI "population stud*" or AB "population stud*" \| Search modes - Find all my search terms \| 5,282 \| \| S90 \| TI prospective or AB prospective \| Search modes - Find all my search terms \| 208,260 \| \| S91 \| TI longitudinal or AB longitudinal \| Search modes - Find all my search terms \| 104,173 \| \| S92 \| TI ("multi dimensional" or multidimensional) or AB ("multi dimensional" or multidimensional) \| Search modes - Find all my search terms \| 15,192 \| \| S93 \| TI "natural experiment*" or AB "natural experiment*" \| Search modes - Find all my search terms \| 1,149 \| \| S94 \| TI ("non random*" or nonrandom*) or AB ("non random*" or nonrandom*) \| Search modes - Find all my search terms \| 11,731 \| \| S95 \| TI ("quasi random*" or quasirandom*) or AB ("quasi random*" or quasirandom*) \| Search modes - Find all my search terms \| 2,185 \| \| S96 \| TI retrospective or AB retrospective \| Search modes - Find all my search terms \| 198,137 \| \| S97 \| S76 OR S77 OR S78 OR S79 OR S80 OR S81 OR S82 OR S83 OR S84 OR S85 OR S86 OR S87 OR S88 OR S89 OR S90 OR S91 OR S92 OR S93 OR S94 OR S95 OR S96 \| Search modes - Find all my search terms \| 2,031,596 \| \| S98 \| S60 AND S97 \| Search modes - Find all my search terms \| 3,916 \| \| S99 \| S75 OR S98 \| Search modes - Find all my search terms \| 4,940 \| \| S100 \| ((MH "Vertebrates+") NOT MH Human) \| Search modes - Find all my search terms \| 213,299 \| \| S101 \| TI (animal* or bovine* or calves or camel* or canine* or cat or cats or chimp* or dog or dogs or equine* or feline* or goat* or hamster* or horse* or llama* or mice* or monkey* or mouse* or pig or piglet* or pigs or porcine* or primate* or rabbit* or rat or rats or rodent* or sheep* or simian* or swine*) \| Search modes - Find all my search terms \| 120,656 \| \| S102 \| S100 OR S101 \| Search modes - Find all my search terms \| 251,330 \| \| S103 \| S99 NOT S102 \| Search modes - Find all my search terms \| 4,912 \| \| S104 \| (MH "Child+") NOT (MH "Adult+" and MH "Child+") \| Search modes - Find all my search terms \| 558,128 \| \| S105 \| TI (adolescen* or child* or infan* or juvenile* or neonat* or paediatric* or pediatric* or youth*) \| Search modes - Find all my search terms \| 595,911 \| \| S106 \| S104 OR S105 \| Search modes - Find all my search terms \| 828,285 \| \| S107 \| S103 NOT S106 \| Search modes - Find all my search terms \| 4,759 \| \| S108 \| (MH "Aged+") NOT ((MH "Adult" or MH "Middle Age" or MH "Young Adult") and MH "Aged+")) \| Search modes - Find all my search terms \| 288,823 \| \| S109 \| TI (aged or elder* or geriatic* or older adult* or senior*) \| Search modes - Find all my search terms \| 134,933 \| \| S110 \| S108 OR S109 \| Search modes - Find all my search terms \| 355,979 \| \| S111 \| S107 NOT S110 \| Search modes - Find all my search terms \| 4,625 \| \| S112 \| (MM "Anesthetics, Local") \| Search modes - Find all my search terms \| 5,771 \| \| S113 \| (MM "Brachial Plexus Block") \| Search modes - Find all my search terms \| 338 \| \| S114 \| TI (anaesth* or anesth* or "brachial plexus block*") \| Search modes - Find all my search terms \| 38,586 \| \| S115 \| S112 OR S113 OR S114 \| Search modes - Find all my search terms \| 41,924 \| \| S116 \| S111 NOT S115 \| Search modes - Find all my search terms \| 4,557 \| \| S117 \| (MM "Arthritis+") \| Search modes - Find all my search terms \| 66,925 \| \| S118 \| TI (arthriti* or osteoarthri* or OA) \| Search modes - Find all my search terms \| 49,993 \| \| S119 \| S117 OR S118 \| Search modes - Find all my search terms \| 76,268 \| \| S120 \| S116 NOT S119 \| Search modes - Find all my search terms \| 4,475 \| \| S121 \| (MM "Breast Neoplasms+") \| Search modes - Find all my search terms \| 77,632 \| \| S122 \| (MM "Head and Neck Neoplasms+") \| Search modes - Find all my search terms \| 49,109 \| \| S123 \| TI cancer* \| Search modes - Find all my search terms \| 321,119 \| \| S124 \| S121 OR S122 OR S123 \| Search modes - Find all my search terms \| 374,828 \| \| S125 \| S120 NOT S124 \| Search modes - Find all my search terms \| 4,342 \| \| S126 \| (MM "Perioperative Care+") \| Search modes - Find all my search terms \| 33,439 \| \| S127 \| TI (intraop* or operative* or periop* or postop*) \| Search modes - Find all my search terms \| 54,696 \| \| S128 \| S126 OR S127 \| Search modes - Find all my search terms \| 77,907 \| \| S129 \| S125 NOT S128 \| Search modes - Find all my search terms \| 4,220 \| \| S130 \| (MM "Shoulder Dislocation") \| Search modes - Find all my search terms \| 1,467 \| \| S131 \| TI (bankart* or dislocat* or instab* or subluxat*) \| Search modes - Find all my search terms \| 14,002 \| \| S132 \| S130 OR S131 \| Search modes - Find all my search terms \| 14,394 \| \| S133 \| S129 NOT S132 \| Search modes - Find all my search terms \| 3,994 \| \| S134 \| (MM "Stroke+") \| Search modes - Find all my search terms \| 58,553 \| \| S135 \| TI (ischaem* or ischem* or or stroke* or TIA) \| Search modes - Find all my search terms \| 15,795 \| \| S136 \| S134 OR S135 \| Search modes - Find all my search terms \| 65,756 \| \| S137 \| S133 NOT S136 \| Search modes - Find all my search terms \| 3,847 \| \| S138 \| TI (editor* or comment* or letter* or news*) \| Search modes - Find all my search terms \| 223,631 \| \| S139 \| S137 NOT S138 \| Search modes - Find all my search terms \| 3,783 \| \| S140 \| S137 NOT S138 \| Limiters - English Language  Search modes - Find all my search terms \| 3,654 \| |
| --- | --- | --- | --- | --- | --- | --- | --- | --- | --- | --- | --- | --- | --- | --- | --- | --- | --- | --- | --- | --- | --- | --- | --- | --- | --- | --- | --- | --- | --- | --- | --- | --- | --- | --- | --- | --- | --- | --- | --- | --- | --- | --- | --- | --- | --- | --- | --- | --- | --- | --- | --- | --- | --- | --- | --- | --- | --- | --- | --- | --- | --- | --- | --- | --- | --- | --- | --- | --- | --- | --- | --- | --- | --- | --- | --- | --- | --- | --- | --- | --- | --- | --- | --- | --- | --- | --- | --- | --- | --- | --- | --- | --- | --- | --- | --- | --- | --- | --- | --- | --- | --- | --- | --- | --- | --- | --- | --- | --- | --- | --- | --- | --- | --- | --- | --- | --- | --- | --- | --- | --- | --- | --- | --- | --- | --- | --- | --- | --- | --- | --- | --- | --- | --- | --- | --- | --- | --- | --- | --- | --- | --- | --- | --- | --- | --- | --- | --- | --- | --- | --- | --- | --- | --- | --- | --- | --- | --- | --- | --- | --- | --- | --- | --- | --- | --- | --- | --- | --- | --- | --- | --- | --- | --- | --- | --- | --- | --- | --- | --- | --- | --- | --- | --- | --- | --- | --- | --- | --- | --- | --- | --- | --- | --- | --- | --- | --- | --- | --- | --- | --- | --- | --- | --- | --- | --- | --- | --- | --- | --- | --- | --- | --- | --- | --- | --- | --- | --- | --- | --- | --- | --- | --- | --- | --- | --- | --- | --- | --- | --- | --- | --- | --- | --- | --- | --- | --- | --- | --- | --- | --- | --- | --- | --- | --- | --- | --- | --- | --- | --- | --- | --- | --- | --- | --- | --- | --- | --- | --- | --- | --- | --- | --- | --- | --- | --- | --- | --- | --- | --- | --- | --- | --- | --- | --- | --- | --- | --- | --- | --- | --- | --- | --- | --- | --- | --- | --- | --- | --- | --- | --- | --- | --- | --- | --- | --- | --- | --- | --- | --- | --- | --- | --- | --- | --- | --- | --- | --- | --- | --- | --- | --- | --- | --- | --- | --- | --- | --- | --- | --- | --- | --- | --- | --- | --- | --- | --- | --- | --- | --- | --- | --- | --- | --- | --- | --- | --- | --- | --- | --- | --- | --- | --- | --- | --- | --- | --- | --- | --- | --- | --- | --- | --- | --- | --- | --- | --- | --- | --- | --- | --- | --- | --- | --- | --- | --- | --- | --- | --- | --- | --- | --- | --- | --- | --- | --- | --- | --- | --- | --- | --- | --- | --- | --- | --- | --- | --- | --- | --- | --- | --- | --- | --- | --- | --- | --- | --- | --- | --- | --- | --- | --- | --- | --- | --- | --- | --- | --- | --- | --- | --- | --- | --- | --- | --- | --- | --- | --- | --- | --- | --- | --- | --- | --- | --- | --- | --- | --- | --- | --- | --- | --- | --- | --- | --- | --- | --- | --- | --- | --- | --- | --- | --- | --- | --- | --- | --- | --- | --- | --- | --- | --- | --- | --- | --- | --- | --- | --- | --- | --- | --- | --- | --- | --- | --- | --- | --- | --- | --- | --- | --- | --- | --- | --- | --- | --- | --- | --- | --- | --- | --- | --- | --- | --- | --- | --- | --- | --- | --- | --- | --- | --- | --- | --- | --- | --- | --- | --- | --- | --- | --- | --- | --- | --- | --- | --- | --- | --- | --- | --- | --- | --- | --- | --- | --- | --- | --- | --- | --- | --- | --- | --- | --- | --- | --- | --- | --- | --- | --- | --- | --- | --- | --- | --- | --- | --- | --- | --- | --- | --- | --- | --- | --- | --- | --- | --- | --- | --- | --- | --- | --- | --- | --- | --- | --- | --- | --- | --- | --- | --- | --- | --- | --- | --- | --- |

**Database: SPORTDiscus with Full Text via EBSCOhost**

**Strategy:**

| \| **#** \| **Query** \| **Limiters/Expanders** \| **Results** \| \| --- \| --- \| --- \| --- \| \| S1 \| DE "BURSITIS" and (TI (gleno* or "rotator cuff*" or shoulder*) or AB (gleno* or "rotator cuff*" or shoulder*)) \| Search modes - Find all my search terms \| 153 \| \| S2 \| DE "ROTATOR cuff" \| Search modes - Find all my search terms \| 1,461 \| \| S3 \| DE "ROTATOR cuff -- Wounds & injuries" \| Search modes - Find all my search terms \| 4,404 \| \| S4 \| DE "SCAPULA" \| Search modes - Find all my search terms \| 1,146 \| \| S5 \| DE "SHOULDER" \| Search modes - Find all my search terms \| 4,325 \| \| S6 \| DE "SHOULDER injuries" \| Search modes - Find all my search terms \| 1,884 \| \| S7 \| DE "SHOULDER joint" \| Search modes - Find all my search terms \| 1,980 \| \| S8 \| DE "SHOULDER joint injuries" \| Search modes - Find all my search terms \| 260 \| \| S9 \| DE "SHOULDER pain" \| Search modes - Find all my search terms \| 1,178 \| \| S10 \| DE "SYNOVITIS" and (TI (gleno* or "rotator cuff*" or shoulder*) or AB (gleno* or "rotator cuff*" or shoulder*)) \| Search modes - Find all my search terms \| 14 \| \| S11 \| DE "TENDINITIS" and (TI (gleno* or "rotator cuff*" or shoulder*) or AB (gleno* or "rotator cuff*" or shoulder*)) \| Search modes - Find all my search terms \| 436 \| \| S12 \| DE "TENDINOSIS" and (TI (gleno* or "rotator cuff*" or shoulder*) or AB (gleno* or "rotator cuff*" or shoulder*)) \| Search modes - Find all my search terms \| 36 \| \| S13 \| TI ((acromion* or coracoid* or gleno* or "infra spinatus*" or infraspinatus* or "rotator cuff*" or shoulder* or scapul* or "supra spinatus*" or supraspinatus* or "teres minor*") N3 (bursiti* or capsuliti* or degenerati* or disease* or disorder* or imping* or inflam* or injur* or pain* or patholog* or problem* or ruptur* or stiff* or strain* or stress* or tear* or tendin* or tendoni* or tendono* or tenosyno* or torn* or trauma* or weak* or wear*)) or AB ((acromion* or coracoid* or gleno* or "infra spinatus*" or infraspinatus* or "rotator cuff*" or shoulder* or scapul* or "supra spinatus*" or supraspinatus* or "teres minor*") N3 (bursiti* or capsuliti* or degenerati* or disease* or disorder* or imping* or inflam* or injur* or pain* or patholog* or problem* or ruptur* or stiff* or strain* or stress* or tear* or tendin* or tendoni* or tendono* or tenosyno* or torn* or trauma* or weak* or wear*)) \| Search modes - Find all my search terms \| 8,935 \| \| S14 \| TI "frozen shoulder*" or AB "frozen shoulder*" \| Search modes - Find all my search terms \| 243 \| \| S15 \| TI "SLAP tear*" or AB "SLAP tear*" \| Search modes - Find all my search terms \| 62 \| \| S16 \| S1 OR S2 OR S3 OR S4 OR S5 OR S6 OR S7 OR S8 OR S9 OR S10 OR S11 OR S12 OR S13 OR S14 OR S15 \| Search modes - Find all my search terms \| 15,286 \| \| S17 \| DE "ACUPRESSURE" \| Search modes - Find all my search terms \| 257 \| \| S18 \| DE "ACUPUNCTURE" \| Search modes - Find all my search terms \| 997 \| \| S19 \| DE "ADRENOCORTICAL hormones" \| Search modes - Find all my search terms \| 988 \| \| S20 \| DE "ANTI-inflammatory agents" \| Search modes - Find all my search terms \| 1,094 \| \| S21 \| DE "COLD therapy" \| Search modes - Find all my search terms \| 847 \| \| S22 \| DE "ELECTRIC stimulation" \| Search modes - Find all my search terms \| 4,109 \| \| S23 \| DE "EXERCISE therapy" \| Search modes - Find all my search terms \| 6,878 \| \| S24 \| DE "GLUCOCORTICOIDS" \| Search modes - Find all my search terms \| 570 \| \| S25 \| DE "INJECTIONS" \| Search modes - Find all my search terms \| 1,319 \| \| S26 \| DE "MEDICAL rehabilitation" \| Search modes - Find all my search terms \| 4,522 \| \| S27 \| DE "NONSTEROIDAL anti-inflammatory agents" \| Search modes - Find all my search terms \| 1,001 \| \| S28 \| DE "PHYSICAL medicine" \| Search modes - Find all my search terms \| 992 \| \| S29 \| DE "PHYSICIANS (General practice)" \| Search modes - Find all my search terms \| 1,424 \| \| S30 \| DE "POSTURE" \| Search modes - Find all my search terms \| 9,524 \| \| S31 \| TI (acupunctur* or acupressure*) or AB (acupunctur* or acupressure*) \| Search modes - Find all my search terms \| 1,527 \| \| S32 \| TI ((adjust* or intervention* or modif* or stabil*) N2 postur*) or AB ((adjust* or intervention* or modif* or stabil*) N2 postur*) \| Search modes - Find all my search terms \| 2,392 \| \| S33 \| TI ("anti inflammator*" or antiinflammator* or NSAID*) or AB ("anti inflammator*" or antiinflammator* or NSAID*) \| Search modes - Find all my search terms \| 4,585 \| \| S34 \| TI ((appl* or pack* or pad*) N2 (hot or heat*)) or AB ((appl* or pack* or pad*) N2 (hot or heat*)) \| Search modes - Find all my search terms \| 481 \| \| S35 \| TI ((appl* or pack or pad*) N2 (ice or cold)) or AB ((appl* or pack or pad*) N2 (ice or cold)) \| Search modes - Find all my search terms \| 605 \| \| S36 \| TI ((care* or healthcare*) N2 primary) or AB ((care* or healthcare*) N2 primary \| Search modes - Find all my search terms \| 4,272 \| \| S37 \| TI chiropract* or AB chiropract* \| Search modes - Find all my search terms \| 2,034 \| \| S38 \| TI ("cold therap*" or cryotherap*) or AB ("cold therap*" or cryotherap*) \| Search modes - Find all my search terms \| 918 \| \| S39 \| TI ((conservative* or "non operative*" or "non surgical*" or nonoperative* or nonsurgical*) N3 (manage* or therap* or treat*)) or AB ((conservative* or "non operative*" or "non surgical*" or nonoperative* or nonsurgical*) N3 (manage* or therap* or treat*)) \| Search modes - Find all my search terms \| 6,029 \| \| S40 \| TI ((cortico* or cortiso* or glucocortico* or prednison* or steroid*) N2 (inject* or shot*)) or AB ((cortico* or cortiso* or glucocortico* or prednison* or steroid*) N2 (inject* or shot*)) \| Search modes - Find all my search terms \| 1,365 \| \| S41 \| TI ((doctor* or physician*) N1 office*) or AB ((doctor* or physician*) N1 office*) \| Search modes - Find all my search terms \| 208 \| \| S42 \| TI "dry needling*" or AB "dry needling*" \| Search modes - Find all my search terms \| 358 \| \| S43 \| TI (exercis* N2 therap*) or AB (exercis* N2 therap*) \| Search modes - Find all my search terms \| 2,888 \| \| S44 \| TI "general practi*" or AB "general practi*" \| Search modes - Find all my search terms \| 1,325 \| \| S45 \| TI icing or AB icing \| Search modes - Find all my search terms \| 238 \| \| S46 \| TI "interferential current*" or AB "interferential current*" \| Search modes - Find all my search terms \| 89 \| \| S47 \| TI "manual therap*" or AB "manual therap*" \| Search modes - Find all my search terms \| 1,533 \| \| S48 \| TI "motion therap*" or AB "motion therap*" \| Search modes - Find all my search terms \| 15 \| \| S49 \| TI ("physio therap*" or physiotherap* or "physical therap*") or AB ("physio therap*" or physiotherap* or "physical therap*") \| Search modes - Find all my search terms \| 23,375 \| \| S50 \| TI rehab* or AB rehab* \| Search modes - Find all my search terms \| 41,381 \| \| S51 \| TI ((resistance or strength* or weight*) N1 train*) or AB (resistance or strength* or weight*) N1 train*) \| Search modes - Find all my search terms \| 22,499 \| \| S52 \| TI (stretches or stretching) or AB (stretches or stretching) \| Search modes - Find all my search terms \| 13,055 \| \| S53 \| TI (wait* N2 watch*) or AB (wait* N2 watch*) \| Search modes - Find all my search terms \| 44 \| \| S54 \| S17 OR S18 OR S19 OR S20 OR S21 OR S22 OR S23 OR S24 OR S25 OR S26 OR S27 OR S28 OR S29 OR S30 OR S31 OR S32 OR S33 OR S34 OR S35 OR S36 OR S37 OR S38 OR S39 OR S40 OR S41 OR S42 OR S43 OR S44 OR S45 OR S46 OR S47 OR S48 OR S49 OR S50 OR S51 OR S52 OR S53 \| Search modes - Find all my search terms \| 136,623 \| \| S55 \| S16 AND S54 \| Search modes - Find all my search terms \| 4,092 \| \| S56 \| TX (allocat* N1 random*) \| Search modes - Find all my search terms \| 5,995 \| \| S57 \| TX ( (doubl* N1 blind*) or (doubl* N1 mask*) ) \| Search modes - Find all my search terms \| 20,119 \| \| S58 \| TX clinic* N1 trial* \| Search modes - Find all my search terms \| 46,522 \| \| S59 \| TX placebo* \| Search modes - Find all my search terms \| 30,087 \| \| S60 \| TX randomi* control* trial* \| Search modes - Find all my search terms \| 76,823 \| \| S61 \| TX ( (singl* N1 blind*) or (singl* N1 mask*) ) \| Search modes - Find all my search terms \| 4,614 \| \| S62 \| TX ( (trebl* N1 blind*) or (trebl* N1 mask*) ) \| Search modes - Find all my search terms \| 2 \| \| S63 \| TX ( (tripl* N1 blind*) or (tripl* N1 mask*) ) \| Search modes - Find all my search terms \| 188 \| \| S64 \| S56 OR S57 OR S58 OR S59 OR S60 OR S61 OR S62 OR S63 \| Search modes - Find all my search terms \| 110,477 \| \| S65 \| S55 AND S64 \| Search modes - Find all my search terms \| 970 \| \| S66 \| TI "case control*" or AB "case control*" \| Search modes - Find all my search terms \| 4,079 \| \| S67 \| TI cohort* or AB cohort* \| Search modes - Find all my search terms \| 23,689 \| \| S68 \| TI "comparative stud*" or AB "comparative stud*" \| Search modes - Find all my search terms \| 3,724 \| \| S69 \| TI (control or controlled) or AB (control or controlled) \| Search modes - Find all my search terms \| 139,543 \| \| S70 \| TI "cross sectional" or AB "cross sectional" \| Search modes - Find all my search terms \| 24,457 \| \| S71 \| TI "descriptive stud*" or AB "descriptive stud*" \| Search modes - Find all my search terms \| 1,278 \| \| S72 \| TI ((design* or studies or study or test*) N2 (post* or pre*)) or AB ((design* or studies or study or test*) N2 (post* or pre*)) \| Search modes - Find all my search terms \| 86,051 \| \| S73 \| TI ("follow up" or followup) or AB ("follow up" or followup) \| Search modes - Find all my search terms \| 34,751 \| \| S74 \| TI observational or AB observational \| Search modes - Find all my search terms \| 8,057 \| \| S75 \| TI "population stud*" or AB "population stud*" \| Search modes - Find all my search terms \| 670 \| \| S76 \| TI prospective or AB prospective \| Search modes - Find all my search terms \| 21,406 \| \| S77 \| TI longitudinal or AB longitudinal \| Search modes - Find all my search terms \| 15,271 \| \| S78 \| TI ("multi dimensional" or multidimensional) or AB ("multi dimensional" or multidimensional) \| Search modes - Find all my search terms \| 3,468 \| \| S79 \| TI "natural experiment*" or AB "natural experiment*" \| Search modes - Find all my search terms \| 171 \| \| S80 \| TI ("non random*" or nonrandom*) or AB ("non random*" or nonrandom*) \| Search modes - Find all my search terms \| 1,218 \| \| S81 \| TI ("quasi random*" or quasirandom*) or AB ("quasi random*" or quasirandom*) \| Search modes - Find all my search terms \| 199 \| \| S82 \| TI retrospective or AB retrospective \| Search modes - Find all my search terms \| 13,193 \| \| S83 \| S66 OR S67 OR S68 OR S69 OR S70 OR S71 OR S72 OR S73 OR S74 OR S75 OR S76 OR S77 OR S78 OR S79 OR S80 OR S81 OR S82 \| Search modes - Find all my search terms \| 286,893 \| \| S84 \| S55 AND S83 \| Search modes - Find all my search terms \| 1,730 \| \| S85 \| S65 OR S84 \| Search modes - Find all my search terms \| 2,006 \| \| S86 \| TI (animal* or bovine* or calves or camel* or canine* or cat or cats or chimp* or dog or dogs or equine* or feline* or goat* or hamster* or horse* or llama* or mice* or monkey* or mouse* or pig or piglet* or pigs or porcine* or primate* or rabbit* or rat or rats or rodent* or sheep* or simian* or swine*) \| Search modes - Find all my search terms \| 42,013 \| \| S87 \| S85 NOT S86 \| Search modes - Find all my search terms \| 1,995 \| \| S88 \| TI (adolescen* or child* or infan* or juvenile* or neonat* or paediatric* or pediatric* or youth*) \| Search modes - Find all my search terms \| 75,691 \| \| S89 \| S87 NOT S88 \| Search modes - Find all my search terms \| 1,961 \| \| S90 \| TI (aged or elder* or geriatic* or older adult* or senior*) \| Search modes - Find all my search terms \| 22,761 \| \| S91 \| S89 NOT S90 \| Search modes - Find all my search terms \| 1,951 \| \| S92 \| TI (anaesth* or anesth* or "brachial plexus block*") \| Search modes - Find all my search terms \| 807 \| \| S93 \| S91 NOT S92 \| Search modes - Find all my search terms \| 1,946 \| \| S94 \| TI (arthriti* or osteoarthri* or OA) \| Search modes - Find all my search terms \| 6,098 \| \| S95 \| S93 NOT S94 \| Search modes - Find all my search terms \| 1,934 \| \| S96 \| TI cancer* \| Search modes - Find all my search terms \| 11,692 \| \| S97 \| S95 NOT S96 \| Search modes - Find all my search terms \| 1,912 \| \| S98 \| TI (intraop* or operative* or periop* or postop*) \| Search modes - Find all my search terms \| 3,465 \| \| S99 \| S97 NOT S98 \| Search modes - Find all my search terms \| 1,877 \| \| S100 \| TI (bankart* or dislocat* or instab* or subluxat*) \| Search modes - Find all my search terms \| 5,840 \| \| S101 \| S99 NOT S100 \| Search modes - Find all my search terms \| 1,728 \| \| S102 \| TI (ischaem* or ischem* or or stroke* or TIA) \| Search modes - Find all my search terms \| 791 \| \| S103 \| S101 NOT S102 \| Search modes - Find all my search terms \| 1,728 \| \| S104 \| TI (editor* or comment* or letter* or news*) \| Search modes - Find all my search terms \| 50,610 \| \| S105 \| S103 NOT S104 \| Search modes - Find all my search terms \| 1,711 \| \| S106 \| S103 NOT S104 \| Limiters - Language: English  Search modes - Find all my search terms \| 1,665 \| |
| --- | --- | --- | --- | --- | --- | --- | --- | --- | --- | --- | --- | --- | --- | --- | --- | --- | --- | --- | --- | --- | --- | --- | --- | --- | --- | --- | --- | --- | --- | --- | --- | --- | --- | --- | --- | --- | --- | --- | --- | --- | --- | --- | --- | --- | --- | --- | --- | --- | --- | --- | --- | --- | --- | --- | --- | --- | --- | --- | --- | --- | --- | --- | --- | --- | --- | --- | --- | --- | --- | --- | --- | --- | --- | --- | --- | --- | --- | --- | --- | --- | --- | --- | --- | --- | --- | --- | --- | --- | --- | --- | --- | --- | --- | --- | --- | --- | --- | --- | --- | --- | --- | --- | --- | --- | --- | --- | --- | --- | --- | --- | --- | --- | --- | --- | --- | --- | --- | --- | --- | --- | --- | --- | --- | --- | --- | --- | --- | --- | --- | --- | --- | --- | --- | --- | --- | --- | --- | --- | --- | --- | --- | --- | --- | --- | --- | --- | --- | --- | --- | --- | --- | --- | --- | --- | --- | --- | --- | --- | --- | --- | --- | --- | --- | --- | --- | --- | --- | --- | --- | --- | --- | --- | --- | --- | --- | --- | --- | --- | --- | --- | --- | --- | --- | --- | --- | --- | --- | --- | --- | --- | --- | --- | --- | --- | --- | --- | --- | --- | --- | --- | --- | --- | --- | --- | --- | --- | --- | --- | --- | --- | --- | --- | --- | --- | --- | --- | --- | --- | --- | --- | --- | --- | --- | --- | --- | --- | --- | --- | --- | --- | --- | --- | --- | --- | --- | --- | --- | --- | --- | --- | --- | --- | --- | --- | --- | --- | --- | --- | --- | --- | --- | --- | --- | --- | --- | --- | --- | --- | --- | --- | --- | --- | --- | --- | --- | --- | --- | --- | --- | --- | --- | --- | --- | --- | --- | --- | --- | --- | --- | --- | --- | --- | --- | --- | --- | --- | --- | --- | --- | --- | --- | --- | --- | --- | --- | --- | --- | --- | --- | --- | --- | --- | --- | --- | --- | --- | --- | --- | --- | --- | --- | --- | --- | --- | --- | --- | --- | --- | --- | --- | --- | --- | --- | --- | --- | --- | --- | --- | --- | --- | --- | --- | --- | --- | --- | --- | --- | --- | --- | --- | --- | --- | --- | --- | --- | --- | --- | --- | --- | --- | --- | --- | --- | --- | --- | --- | --- | --- | --- | --- | --- | --- | --- | --- | --- | --- | --- | --- | --- | --- | --- | --- | --- | --- | --- | --- | --- | --- | --- | --- | --- | --- | --- | --- | --- | --- | --- | --- | --- | --- | --- | --- | --- | --- | --- | --- | --- | --- | --- | --- | --- | --- | --- | --- | --- | --- | --- | --- | --- | --- | --- | --- | --- | --- | --- | --- | --- | --- | --- | --- | --- | --- | --- | --- | --- | --- | --- | --- |

**Database: Conference Proceedings Citation Index – Science (CPCI-S) – 1990-present (Web of Science Core Collection)**

**Strategy:**

| # 29 | [**100**](http://apps.webofknowledge.com.login.ezproxy.library.ualberta.ca/summary.do?product=WOS&doc=1&qid=33&SID=7B1Lh4AZrmarNEfGnMh&search_mode=AdvancedSearch&update_back2search_link_param=yes) | (#27 NOT #28) *AND***LANGUAGE:** (English)  *Indexes=CPCI-S Timespan=1990-2018* |
| --- | --- | --- |
| # 28 | [**50,474**](http://apps.webofknowledge.com.login.ezproxy.library.ualberta.ca/summary.do?product=WOS&doc=1&qid=32&SID=7B1Lh4AZrmarNEfGnMh&search_mode=AdvancedSearch&update_back2search_link_param=yes) | (TI=(ischaem* or ischem* or stroke* or TIA)) *AND***LANGUAGE:** (English)  *Indexes=CPCI-S Timespan=1990-2018* |
| # 27 | [**114**](http://apps.webofknowledge.com.login.ezproxy.library.ualberta.ca/summary.do?product=WOS&doc=1&qid=31&SID=7B1Lh4AZrmarNEfGnMh&search_mode=AdvancedSearch&update_back2search_link_param=yes) | (#25 NOT #26) *AND***LANGUAGE:** (English)  *Indexes=CPCI-S Timespan=1990-2018* |
| # 26 | [**17,600**](http://apps.webofknowledge.com.login.ezproxy.library.ualberta.ca/summary.do?product=WOS&doc=1&qid=30&SID=7B1Lh4AZrmarNEfGnMh&search_mode=AdvancedSearch&update_back2search_link_param=yes) | (TI=(bankart* or dislocat* or instab* or subluxat*)) *AND***LANGUAGE:** (English)  *Indexes=CPCI-S Timespan=1990-2018* |
| # 25 | [**122**](http://apps.webofknowledge.com.login.ezproxy.library.ualberta.ca/summary.do?product=WOS&doc=1&qid=29&SID=7B1Lh4AZrmarNEfGnMh&search_mode=AdvancedSearch&update_back2search_link_param=yes) | (#23 NOT #24) *AND***LANGUAGE:** (English)  *Indexes=CPCI-S Timespan=1990-2018* |
| # 24 | [**21,910**](http://apps.webofknowledge.com.login.ezproxy.library.ualberta.ca/summary.do?product=WOS&doc=1&qid=28&SID=7B1Lh4AZrmarNEfGnMh&search_mode=AdvancedSearch&update_back2search_link_param=yes) | (TI=(intraop* or operative* or periop* or postop*)) *AND***LANGUAGE:** (English)  *Indexes=CPCI-S Timespan=1990-2018* |
| # 23 | [**123**](http://apps.webofknowledge.com.login.ezproxy.library.ualberta.ca/summary.do?product=WOS&doc=1&qid=27&SID=7B1Lh4AZrmarNEfGnMh&search_mode=AdvancedSearch&update_back2search_link_param=yes) | (#21 NOT #22) *AND***LANGUAGE:** (English)  *Indexes=CPCI-S Timespan=1990-2018* |
| # 22 | [**192,963**](http://apps.webofknowledge.com.login.ezproxy.library.ualberta.ca/summary.do?product=WOS&doc=1&qid=25&SID=7B1Lh4AZrmarNEfGnMh&search_mode=AdvancedSearch&update_back2search_link_param=yes) | (TI=cancer*) *AND***LANGUAGE:** (English)  *Indexes=CPCI-S Timespan=1990-2018* |
| # 21 | [**123**](http://apps.webofknowledge.com.login.ezproxy.library.ualberta.ca/summary.do?product=WOS&doc=1&qid=24&SID=7B1Lh4AZrmarNEfGnMh&search_mode=AdvancedSearch&update_back2search_link_param=yes) | (#19 NOT #20) *AND***LANGUAGE:** (English)  *Indexes=CPCI-S Timespan=1990-2018* |
| # 20 | [**32,804**](http://apps.webofknowledge.com.login.ezproxy.library.ualberta.ca/summary.do?product=WOS&doc=1&qid=23&SID=7B1Lh4AZrmarNEfGnMh&search_mode=AdvancedSearch&update_back2search_link_param=yes) | (TI=(arthriti* or osteoarthri* or OA)) *AND***LANGUAGE:** (English)  *Indexes=CPCI-S Timespan=1990-2018* |
| # 19 | [**123**](http://apps.webofknowledge.com.login.ezproxy.library.ualberta.ca/summary.do?product=WOS&doc=1&qid=22&SID=7B1Lh4AZrmarNEfGnMh&search_mode=AdvancedSearch&update_back2search_link_param=yes) | (#17 NOT #18) *AND***LANGUAGE:** (English)  *Indexes=CPCI-S Timespan=1990-2018* |
| # 18 | [**8,554**](http://apps.webofknowledge.com.login.ezproxy.library.ualberta.ca/summary.do?product=WOS&doc=1&qid=21&SID=7B1Lh4AZrmarNEfGnMh&search_mode=AdvancedSearch&update_back2search_link_param=yes) | (TI=(anaesth* or anesth* or "brachial plexus block*")) *AND***LANGUAGE:** (English)  *Indexes=CPCI-S Timespan=1990-2018* |
| # 17 | [**123**](http://apps.webofknowledge.com.login.ezproxy.library.ualberta.ca/summary.do?product=WOS&doc=1&qid=20&SID=7B1Lh4AZrmarNEfGnMh&search_mode=AdvancedSearch&update_back2search_link_param=yes) | (#15 NOT #16) *AND***LANGUAGE:** (English)  *Indexes=CPCI-S Timespan=1990-2018* |
| # 16 | [**83,390**](http://apps.webofknowledge.com.login.ezproxy.library.ualberta.ca/summary.do?product=WOS&doc=1&qid=19&SID=7B1Lh4AZrmarNEfGnMh&search_mode=AdvancedSearch&update_back2search_link_param=yes) | (TI=(aged or elder* or geriatic* or older adult* or senior*)) *AND***LANGUAGE:** (English)  *Indexes=CPCI-S Timespan=1990-2018* |
| # 15 | [**123**](http://apps.webofknowledge.com.login.ezproxy.library.ualberta.ca/summary.do?product=WOS&doc=1&qid=18&SID=7B1Lh4AZrmarNEfGnMh&search_mode=AdvancedSearch&update_back2search_link_param=yes) | (#13 NOT #14) *AND***LANGUAGE:** (English)  *Indexes=CPCI-S Timespan=1990-2018* |
| # 14 | [**137,265**](http://apps.webofknowledge.com.login.ezproxy.library.ualberta.ca/summary.do?product=WOS&doc=1&qid=17&SID=7B1Lh4AZrmarNEfGnMh&search_mode=AdvancedSearch&update_back2search_link_param=yes) | (TI=(adolescen* or child* or infan* or juvenile* or neonat* or paediatric* or pediatric* or youth*)) *AND***LANGUAGE:** (English)  *Indexes=CPCI-S Timespan=1990-2018* |
| # 13 | [**124**](http://apps.webofknowledge.com.login.ezproxy.library.ualberta.ca/summary.do?product=WOS&doc=1&qid=16&SID=7B1Lh4AZrmarNEfGnMh&search_mode=AdvancedSearch&update_back2search_link_param=yes) | (#11 NOT #12) *AND***LANGUAGE:** (English)  *Indexes=CPCI-S Timespan=1990-2018* |
| # 12 | [**252,765**](http://apps.webofknowledge.com.login.ezproxy.library.ualberta.ca/summary.do?product=WOS&doc=1&qid=15&SID=7B1Lh4AZrmarNEfGnMh&search_mode=AdvancedSearch&update_back2search_link_param=yes) | (TI=(animal* or bovine* or calves or camel* or canine* or cat or cats or chimp* or dog or dogs or equine* or feline* or goat* or hamster* or horse* or llama* or mice* or monkey* or mouse* or pig or piglet* or pigs or porcine* or primate* or rabbit* or rat or rats or rodent* or sheep* or simian* or swine*)) *AND***LANGUAGE:** (English)  *Indexes=CPCI-S Timespan=1990-2018* |
| # 11 | [**124**](http://apps.webofknowledge.com.login.ezproxy.library.ualberta.ca/summary.do?product=WOS&doc=1&qid=13&SID=7B1Lh4AZrmarNEfGnMh&search_mode=AdvancedSearch&update_back2search_link_param=yes) | (#8 OR #10) *AND***LANGUAGE:** (English)  *Indexes=CPCI-S Timespan=1990-2018* |
| # 10 | [**118**](http://apps.webofknowledge.com.login.ezproxy.library.ualberta.ca/summary.do?product=WOS&doc=1&qid=12&SID=7B1Lh4AZrmarNEfGnMh&search_mode=AdvancedSearch&update_back2search_link_param=yes) | (#6 AND #9) *AND***LANGUAGE:** (English)  *Indexes=CPCI-S Timespan=1990-2018* |
| # 9 | [**1,486,782**](http://apps.webofknowledge.com.login.ezproxy.library.ualberta.ca/summary.do?product=WOS&doc=1&qid=11&SID=7B1Lh4AZrmarNEfGnMh&search_mode=AdvancedSearch&update_back2search_link_param=yes) | (TS=("case control*" or cohort* or "comparative stud*" or control or controlled or "cross sectional" or "descriptive stud*" or ((design* or studies or study or test*) NEAR/2 (post* or pre*)) or "follow up" or followup or observational or "population stud*" or prospective or longitudinal or "multi dimensional" or multidimensional or "natural experiment*" or "non random*" or nonrandom* or "quasi random*" or quasirandom* or retrospective))*AND***LANGUAGE:** (English)  *Indexes=CPCI-S Timespan=1990-2018* |
| # 8 | [**47**](http://apps.webofknowledge.com.login.ezproxy.library.ualberta.ca/summary.do?product=WOS&doc=1&qid=10&SID=7B1Lh4AZrmarNEfGnMh&search_mode=AdvancedSearch&update_back2search_link_param=yes) | (#6 AND #7) *AND***LANGUAGE:** (English)  *Indexes=CPCI-S Timespan=1990-2018* |
| # 7 | [**66,836**](http://apps.webofknowledge.com.login.ezproxy.library.ualberta.ca/summary.do?product=WOS&doc=1&qid=9&SID=7B1Lh4AZrmarNEfGnMh&search_mode=AdvancedSearch&update_back2search_link_param=yes) | (TS=((allocat* NEAR/1 random*) or (doubl* NEAR/1 blind*) or (doubl* NEAR/1 mask*) or (clinic* NEAR/1 trial*) or placebo* or "randomi* control* trial*" or (singl* NEAR/1 blind*) or (singl* NEAR/1 mask*) or (trebl* NEAR/1 blind*) or (trebl* NEAR/1 mask*) or (tripl* NEAR/1 blind*) or (tripl* NEAR/1 mask*)))*AND***LANGUAGE:** (English)  *Indexes=CPCI-S Timespan=1990-2018* |
| # 6 | [**244**](http://apps.webofknowledge.com.login.ezproxy.library.ualberta.ca/summary.do?product=WOS&doc=1&qid=7&SID=7B1Lh4AZrmarNEfGnMh&search_mode=AdvancedSearch&update_back2search_link_param=yes) | (#4 AND #5) *AND***LANGUAGE:** (English)  *Indexes=CPCI-S Timespan=1990-2018* |
| # 5 | [**111,193**](http://apps.webofknowledge.com.login.ezproxy.library.ualberta.ca/summary.do?product=WOS&doc=1&qid=6&SID=7B1Lh4AZrmarNEfGnMh&search_mode=AdvancedSearch&update_back2search_link_param=yes) | (TS=(acupunctur* or acupressure* or ((adjust* or intervention* or modif* or stabil*) NEAR/2 postur*) or "anti inflammator*" or antiinflammator* or NSAID* or ((appl* or pack* or pad*) NEAR/2 (hot or heat*)) or ((appl* or pack or pad*) NEAR/2 (ice or cold)) or ((care* or healthcare*) NEAR/2 primary) or chiropract* or "cold therap*" or cryotherap* or ((conservative* or "non operative*" or "non surgical*" or nonoperative* or nonsurgical*) NEAR/3 (manage* or therap* or treat*)) or ((cortico* or cortiso* or glucocortico* or prednison* or steroid*) NEAR/2 (inject* or shot*)) or ((doctor* or physician*) NEAR/1 office*) or "dry needling*" or (exercis* NEAR/2 therap*) or "general practi*" or icing or "interferential current*" or "manual therap*" or "motion therap*" or "physio therap*" or physiotherap* or "physical therap*" or rehab* or ((resistance or strength* or weight*) NEAR/1 train*) or stretches or stretching or (wait* NEAR/2 watch*))) *AND***LANGUAGE:** (English)  *Indexes=CPCI-S Timespan=1990-2018* |
| # 4 | [**1,293**](http://apps.webofknowledge.com.login.ezproxy.library.ualberta.ca/summary.do?product=WOS&doc=1&qid=4&SID=7B1Lh4AZrmarNEfGnMh&search_mode=AdvancedSearch&update_back2search_link_param=yes) | (#1 OR #2 OR #3) *AND***LANGUAGE:** (English)  *Indexes=CPCI-S Timespan=1990-2018* |
| # 3 | [**2**](http://apps.webofknowledge.com.login.ezproxy.library.ualberta.ca/summary.do?product=WOS&doc=1&qid=3&SID=7B1Lh4AZrmarNEfGnMh&search_mode=AdvancedSearch&update_back2search_link_param=yes) | (TS="SLAP tear*") *AND***LANGUAGE:** (English)  *Indexes=CPCI-S Timespan=1990-2018* |
| # 2 | [**57**](http://apps.webofknowledge.com.login.ezproxy.library.ualberta.ca/summary.do?product=WOS&doc=1&qid=2&SID=7B1Lh4AZrmarNEfGnMh&search_mode=AdvancedSearch&update_back2search_link_param=yes) | (TS="frozen shoulder*") *AND***LANGUAGE:** (English)  *Indexes=CPCI-S Timespan=1990-2018* |
| # 1 | [**1,259**](http://apps.webofknowledge.com.login.ezproxy.library.ualberta.ca/summary.do?product=WOS&doc=1&qid=1&SID=7B1Lh4AZrmarNEfGnMh&search_mode=AdvancedSearch&update_back2search_link_param=yes) | (TS=((acromion* or coracoid* or gleno* or "infra spinatus*" or infraspinatus* or "rotator cuff*" or shoulder* or scapul* or "supra spinatus*" or supraspinatus* or "teres minor*") NEAR/3 (bursiti* or capsuliti* or degenerati* or disease* or disorder* or imping* or inflam* or injur* or pain* or patholog* or problem* or ruptur* or stiff* or strain* or stress* or tear* or tendin* or tendoni* or tendono* or tenosyno* or torn* or trauma* or weak* or wear*))) *AND***LANGUAGE:** (English)  *Indexes=CPCI-S Timespan=1990-2018* |

**Summary of Association Website Search**

| **Website** | **Search method** | **# of records found** | **Note** |
| --- | --- | --- | --- |
| Canadian Academy of Sport and Exercise Medicine | Searched the website using keywords for shoulder term | 1 | “Kocher 1998” found through searching bibliography of “snowboarding injuries” under the section “past CASEM statements” |
| Canadian Athletic Therapists Association |  |  | Login username/ password is needed to access papers |
| Canadian Physiotherapy Association | Searched the website using keywords for shoulder term  Searched the publication section | 0 |  |
| College of Family Physicians of Canada – Sport & Exercise Committee | Searched the website using keywords for shoulder term  Searched the publication section | 0 |  |
| Exercise is Medicine Canada | Searched the website using keywords for shoulder term  Searched with “publication” term | 0 |  |
| Exercise is Medicine Canada | Searched the website using keywords for shoulder term  Searched through “public resources” section | 0 | The site contains several blog posts related when using the keywords search, I did not see any reported studies |
| Ontario Medical Association – Section on Sport & Exercise Medicine | Searched the website using keywords for shoulder term  Searched with “publication” term |  | Not available to public |
| Sport Physiotherapy Canada | Searched the website using keywords for shoulder term  Searched with “publication” term | 0 |  |
