# Supplementary figures and images for: Shoulder specific exercise therapy is effective in reducing chronic shoulder pain: A network meta-analysis
Source: PLoS One. 2024 Apr 29;19(4):e0294014. doi: 10.1371/journal.pone.0294014 (PMC11057978; doi:10.1371/journal.pone.0294014)

# DESCRIPTION OF INTERVENTIONS BY STUDY


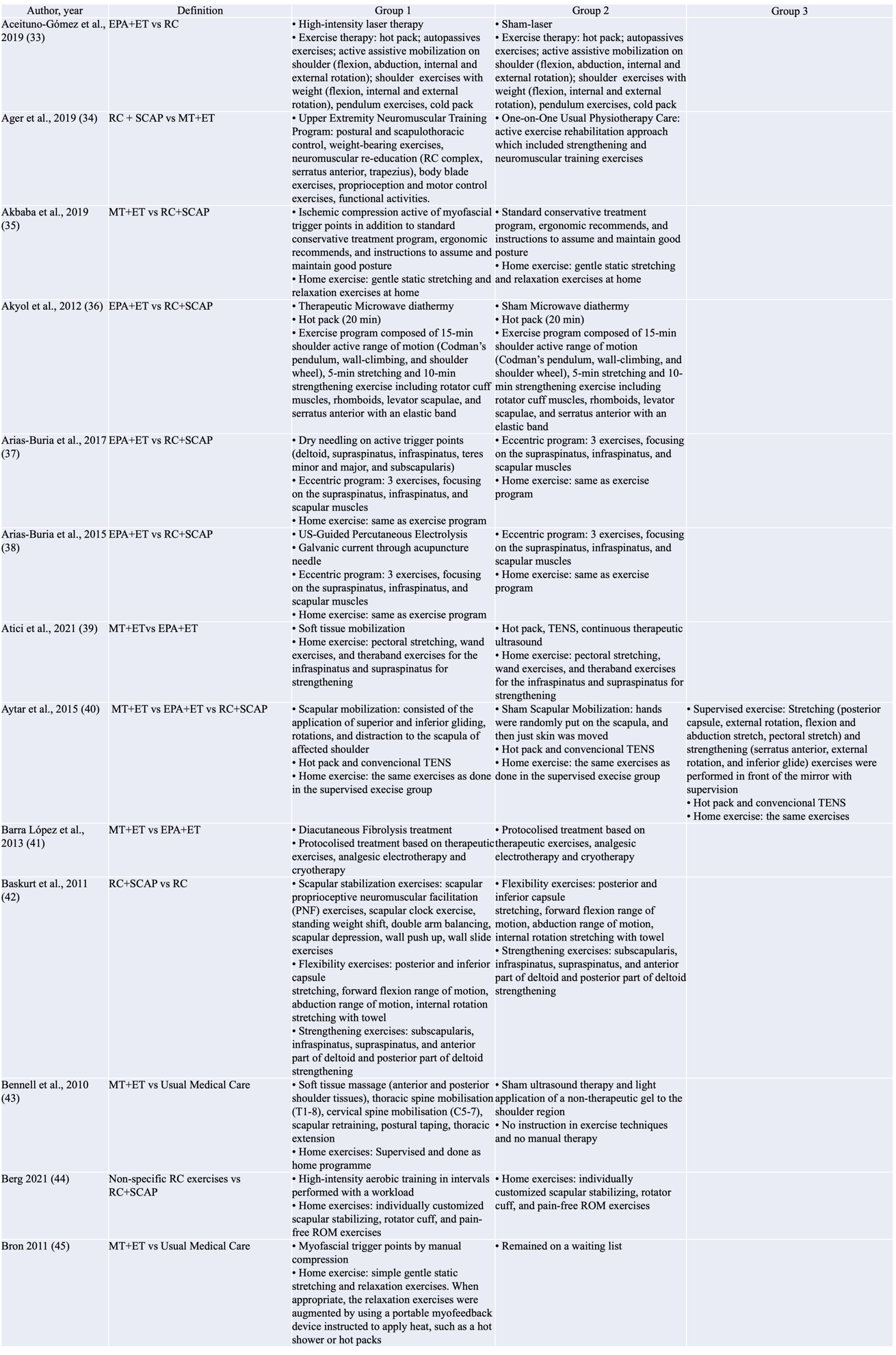

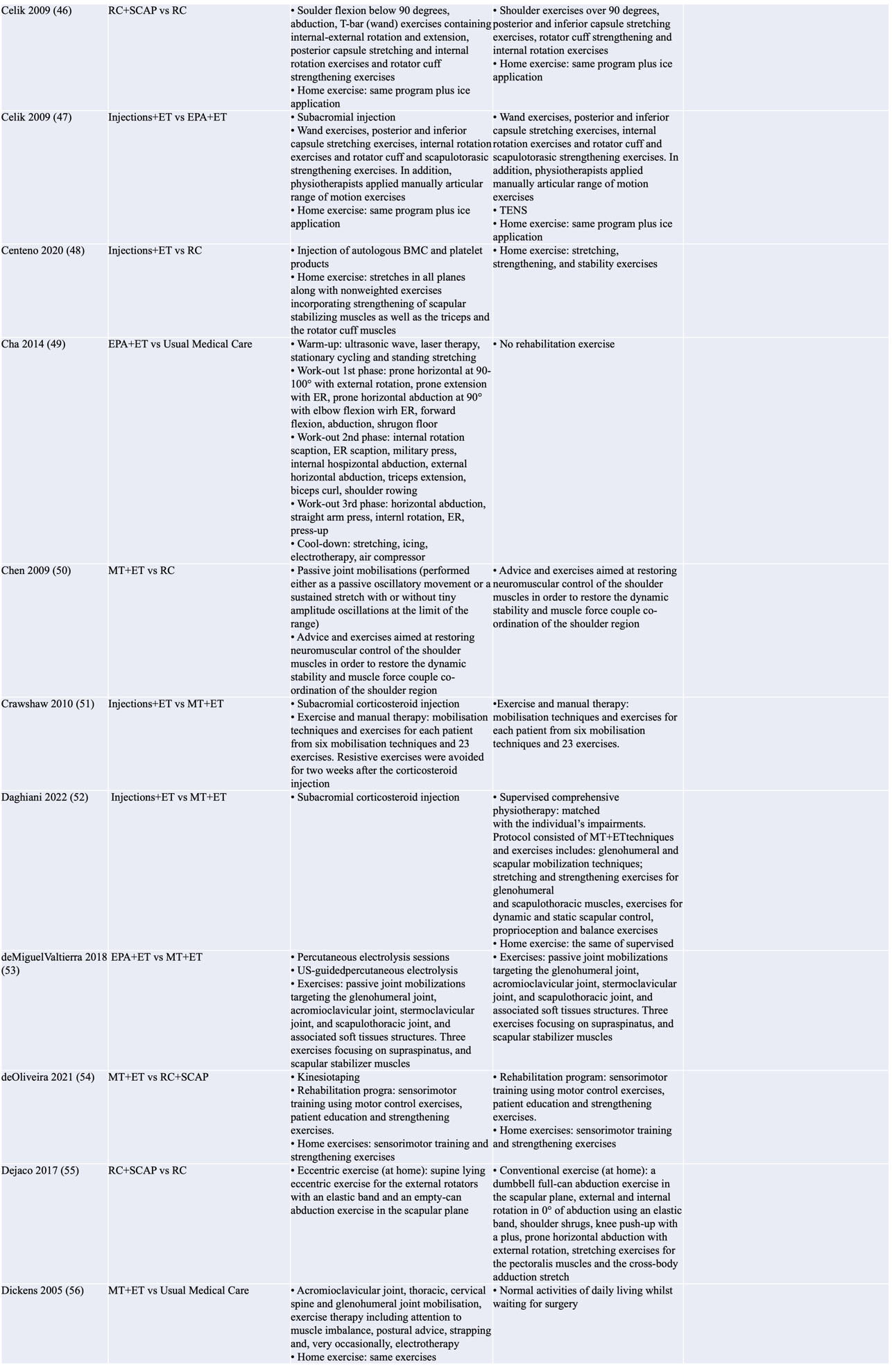

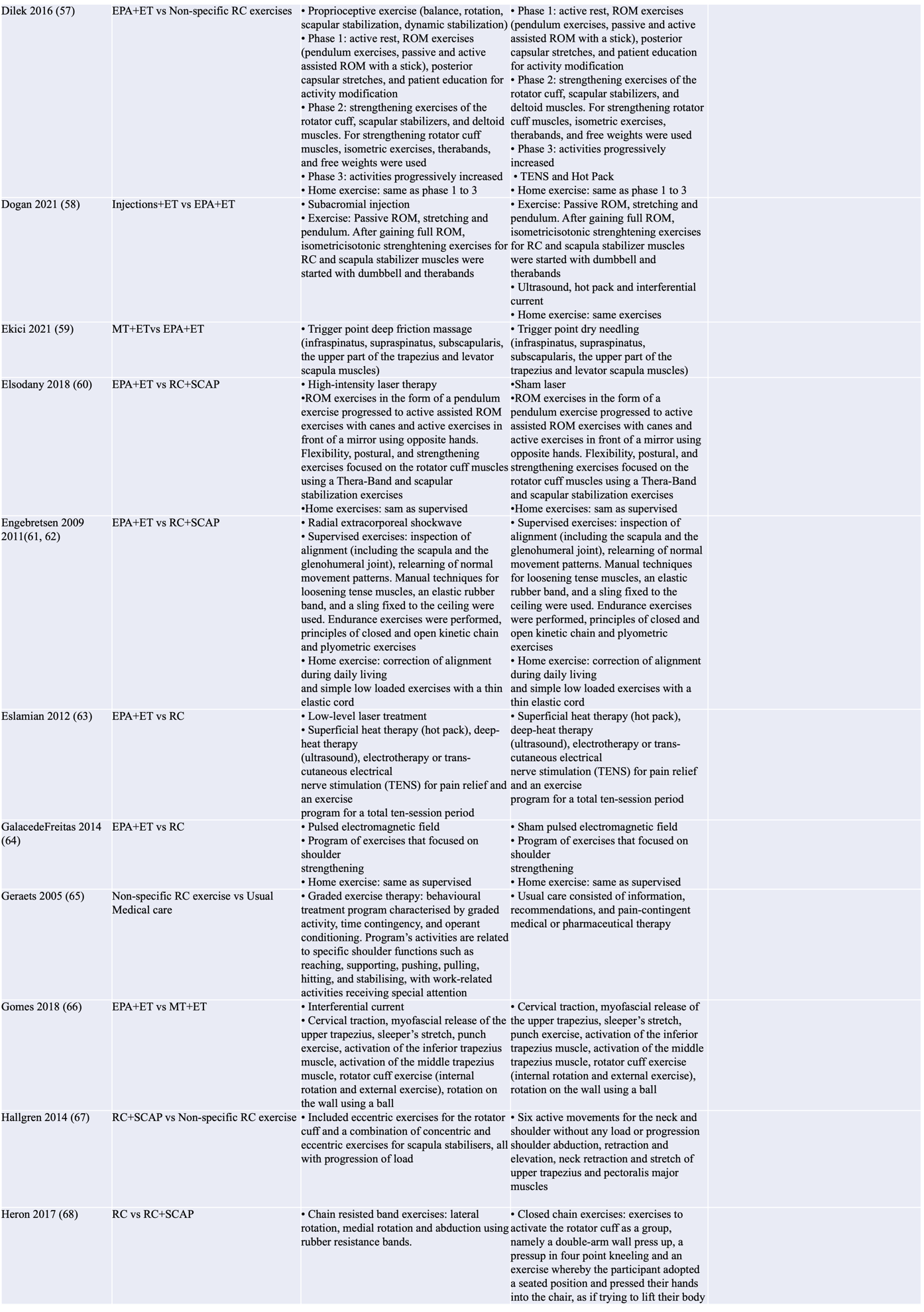

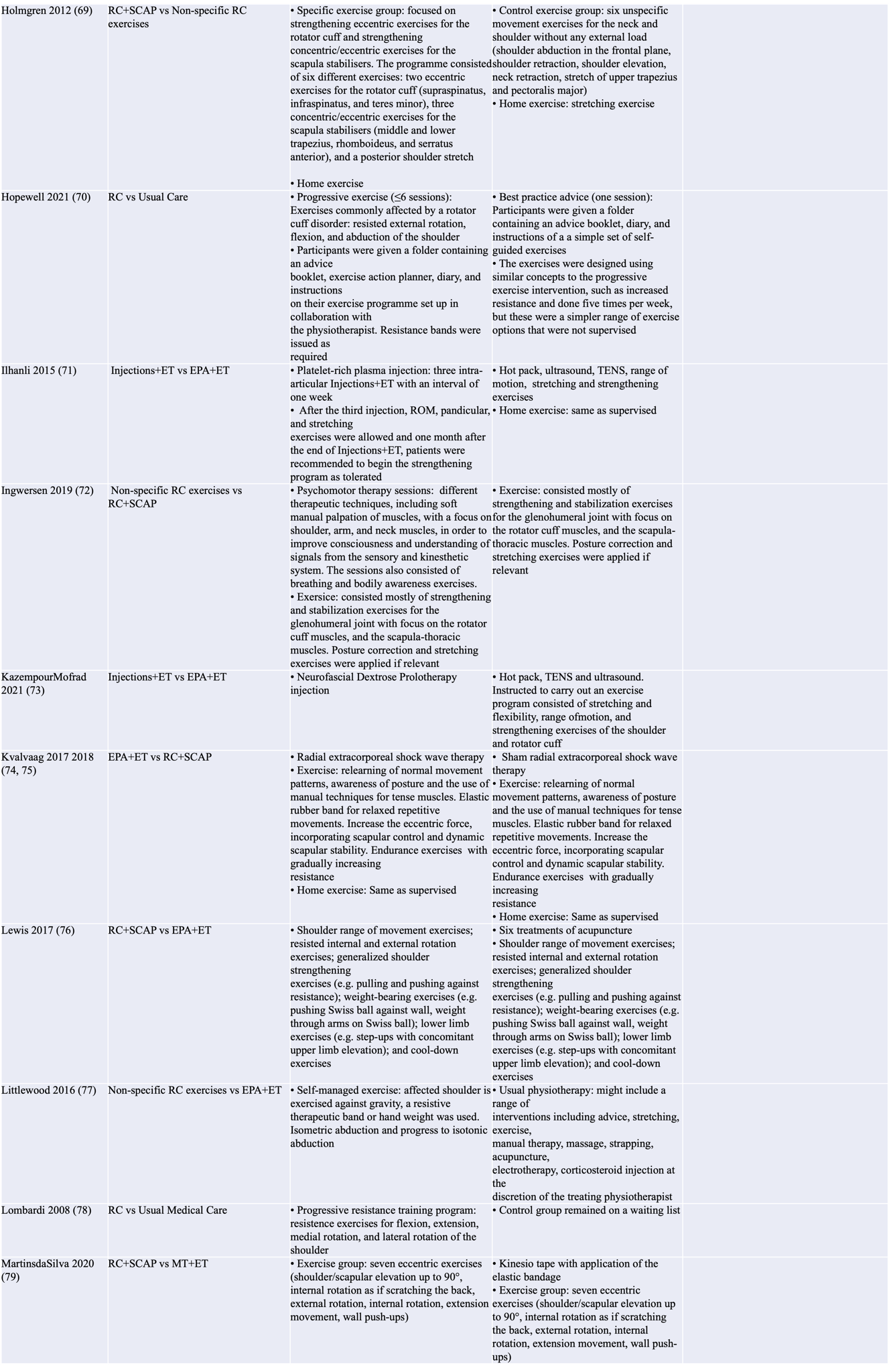

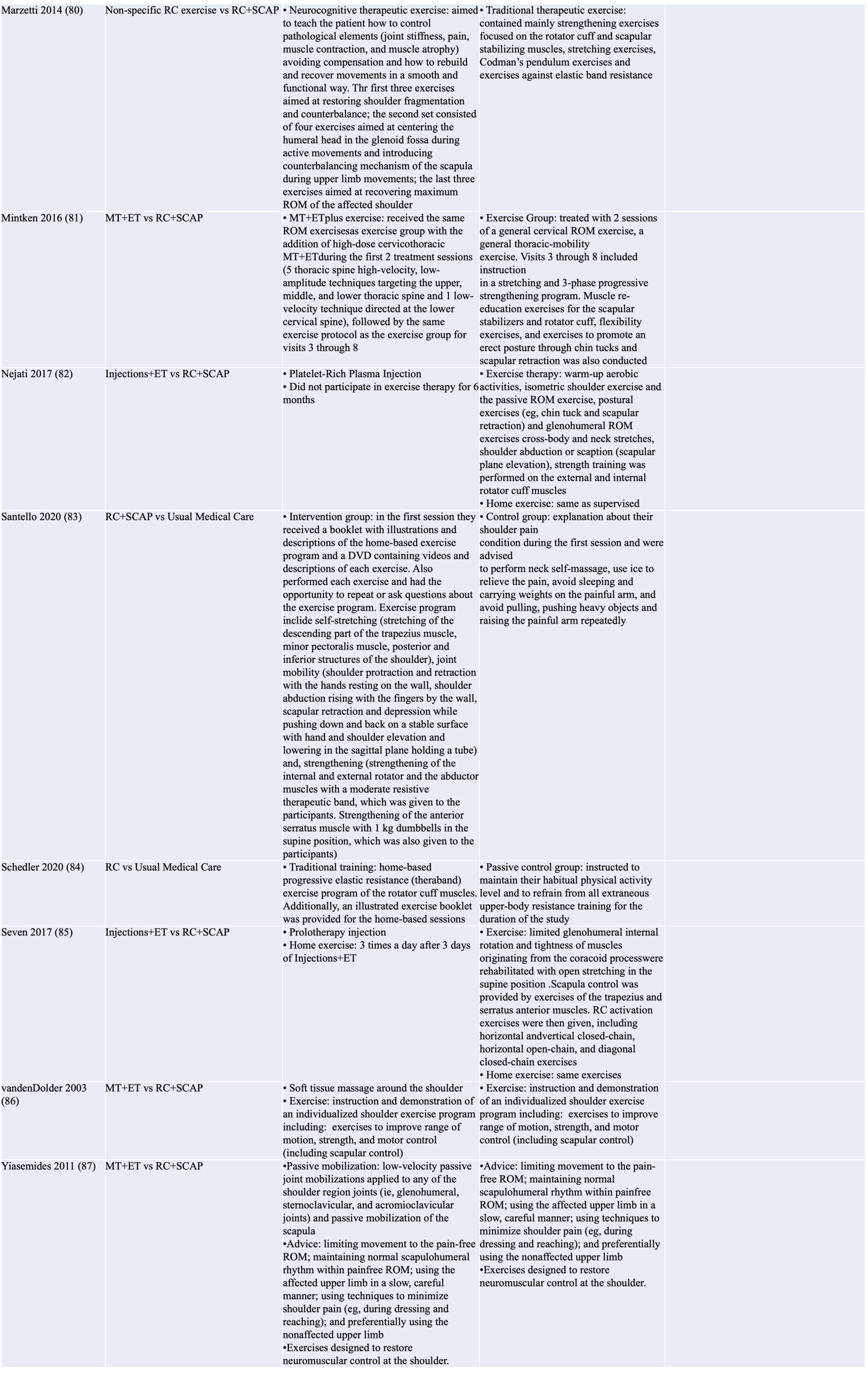

Supplement: S3 Appendix — (DOCX) [file pone.0294014.s003.docx]

**PUBLICATION BIAS TABLES BY OUTCOMES**

#

**PAIN**
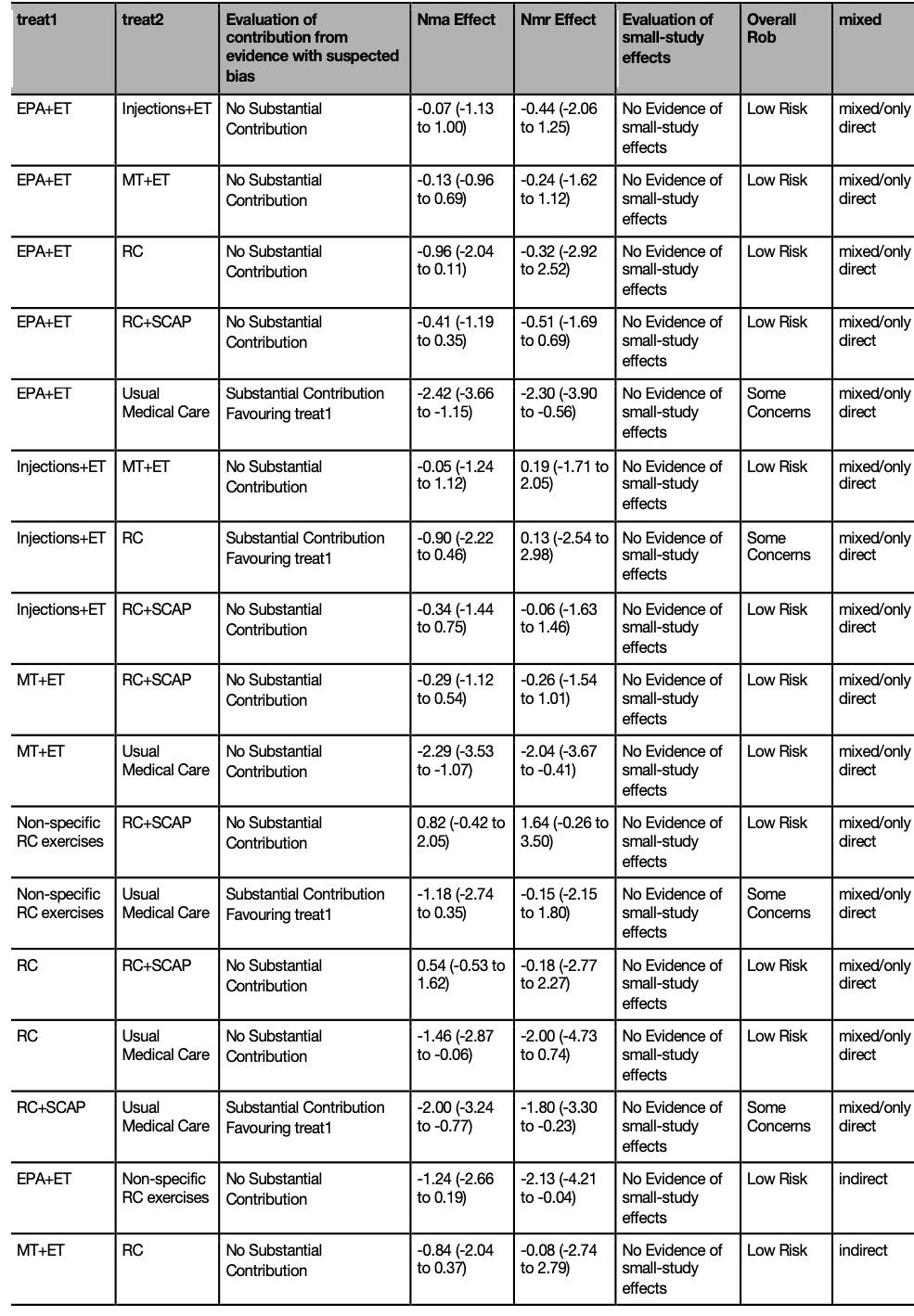


**ROM_ABD**


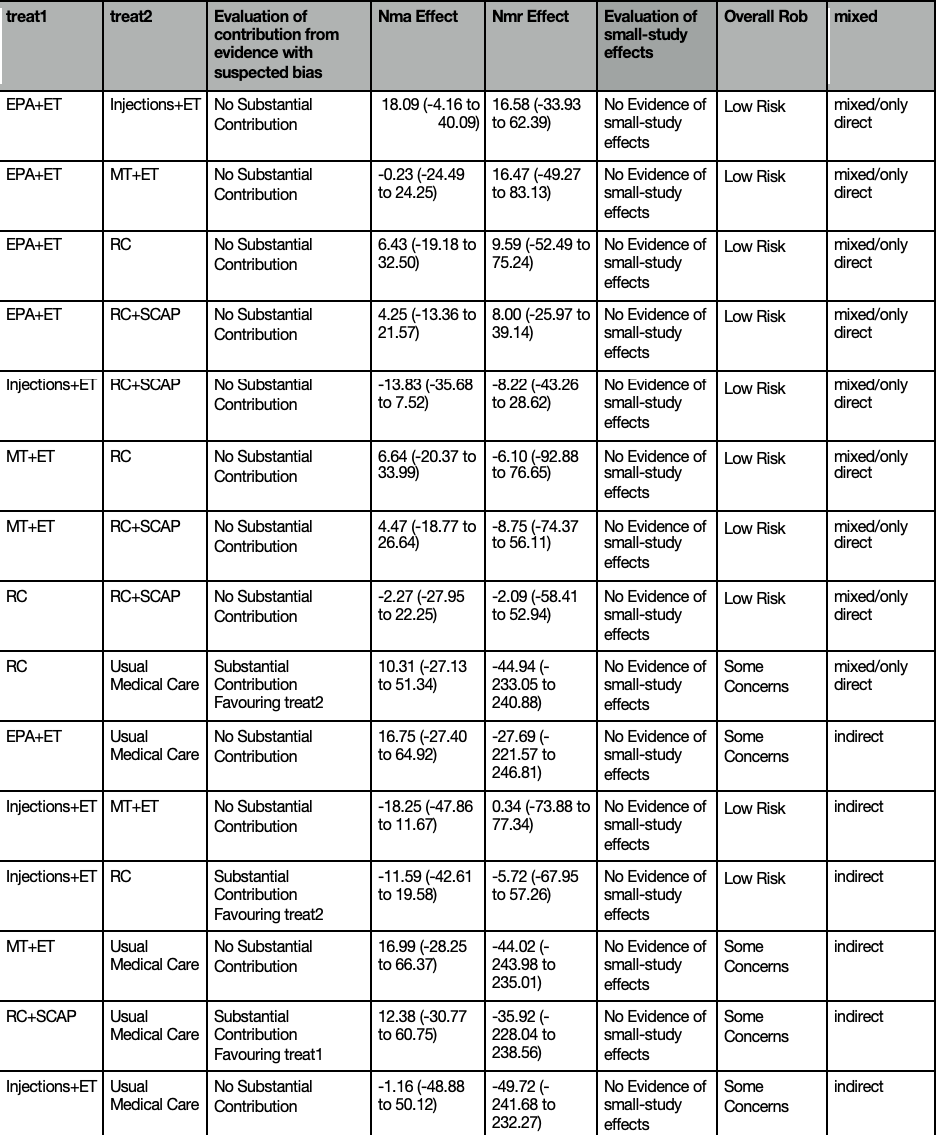


# ROM_ER


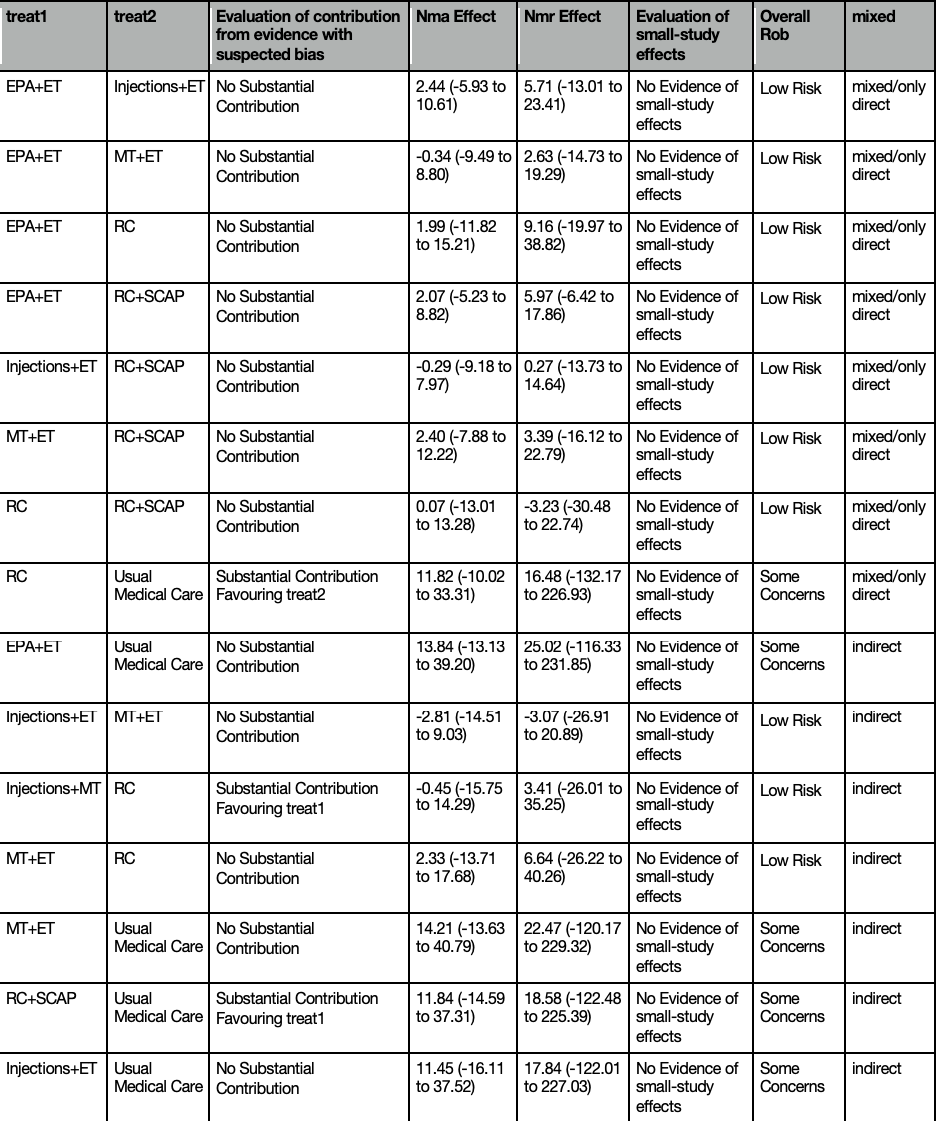


**SPADI**


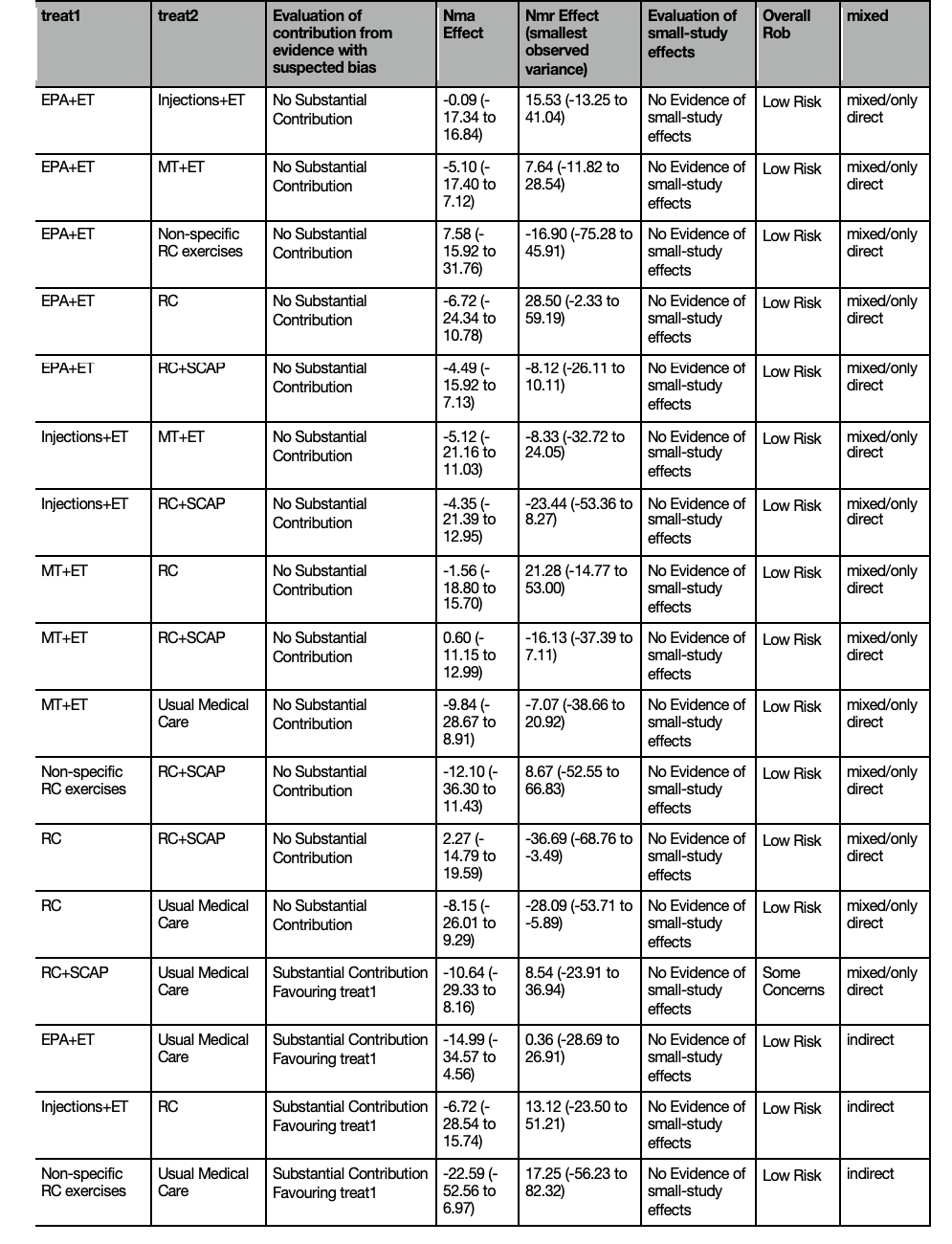


**DASH**
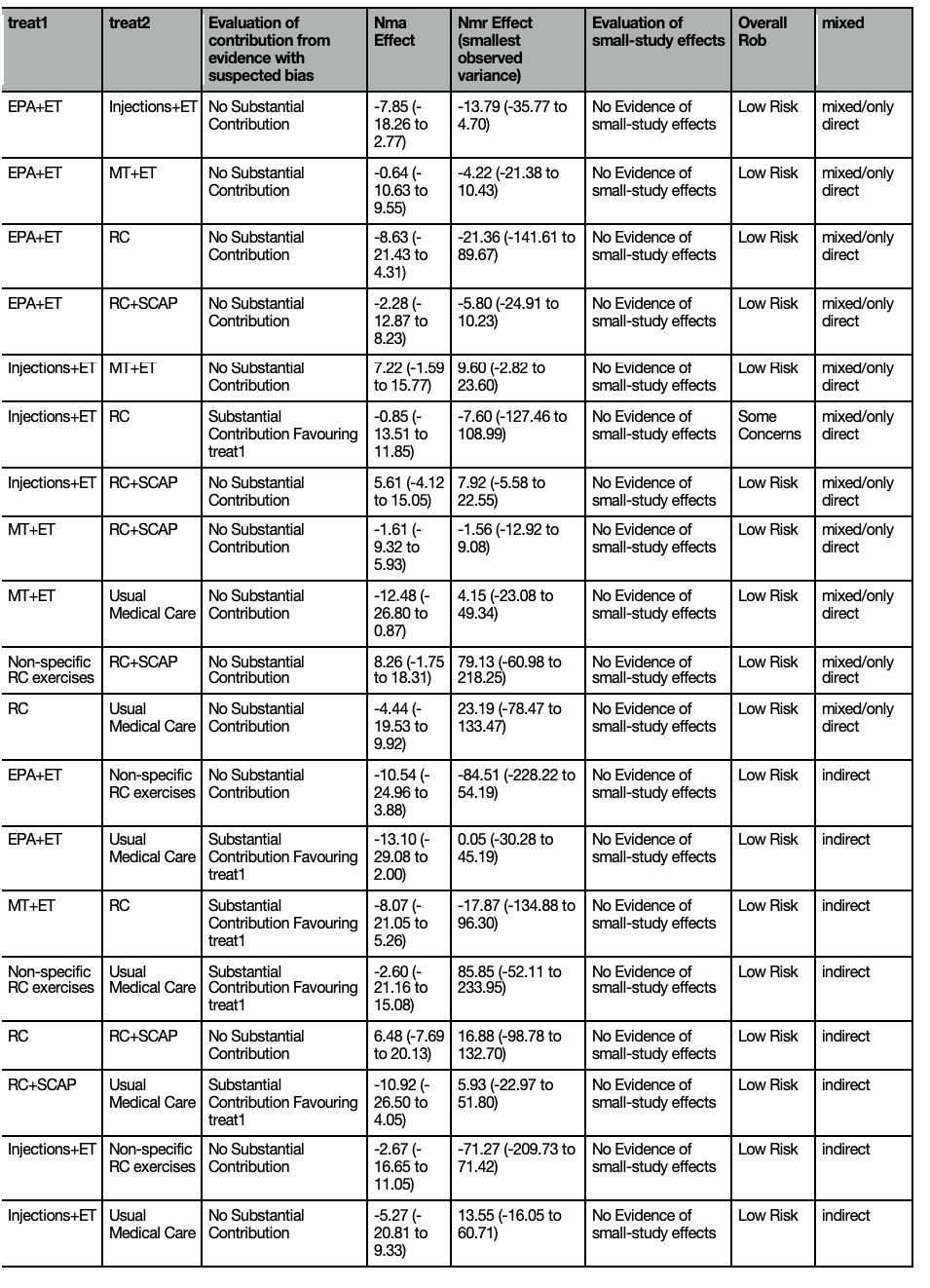

Supplement: S5 Appendix — (DOCX) [file pone.0294014.s005.docx]

# POST-INTERVENTION SENSITIVITY ANALYSIS: PAIN, ROM_ABD, HRQL (SPADI, DASH)


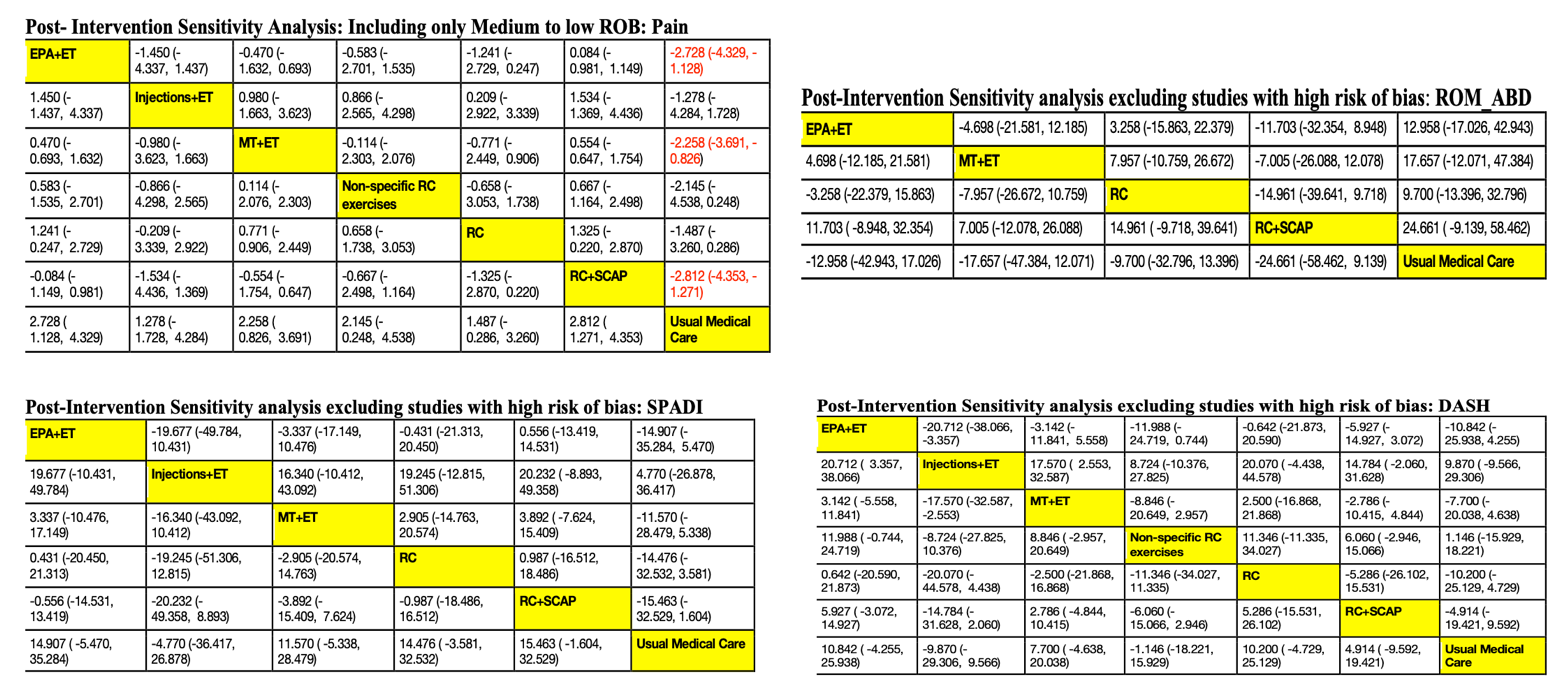

Supplement: S6 Appendix — (DOCX) [file pone.0294014.s006.docx]

**CONFIDENCE IN RESULTS: ROM_ER, ROM_ABD, HRQL (SPADI, DASH)**

# ROM_ER


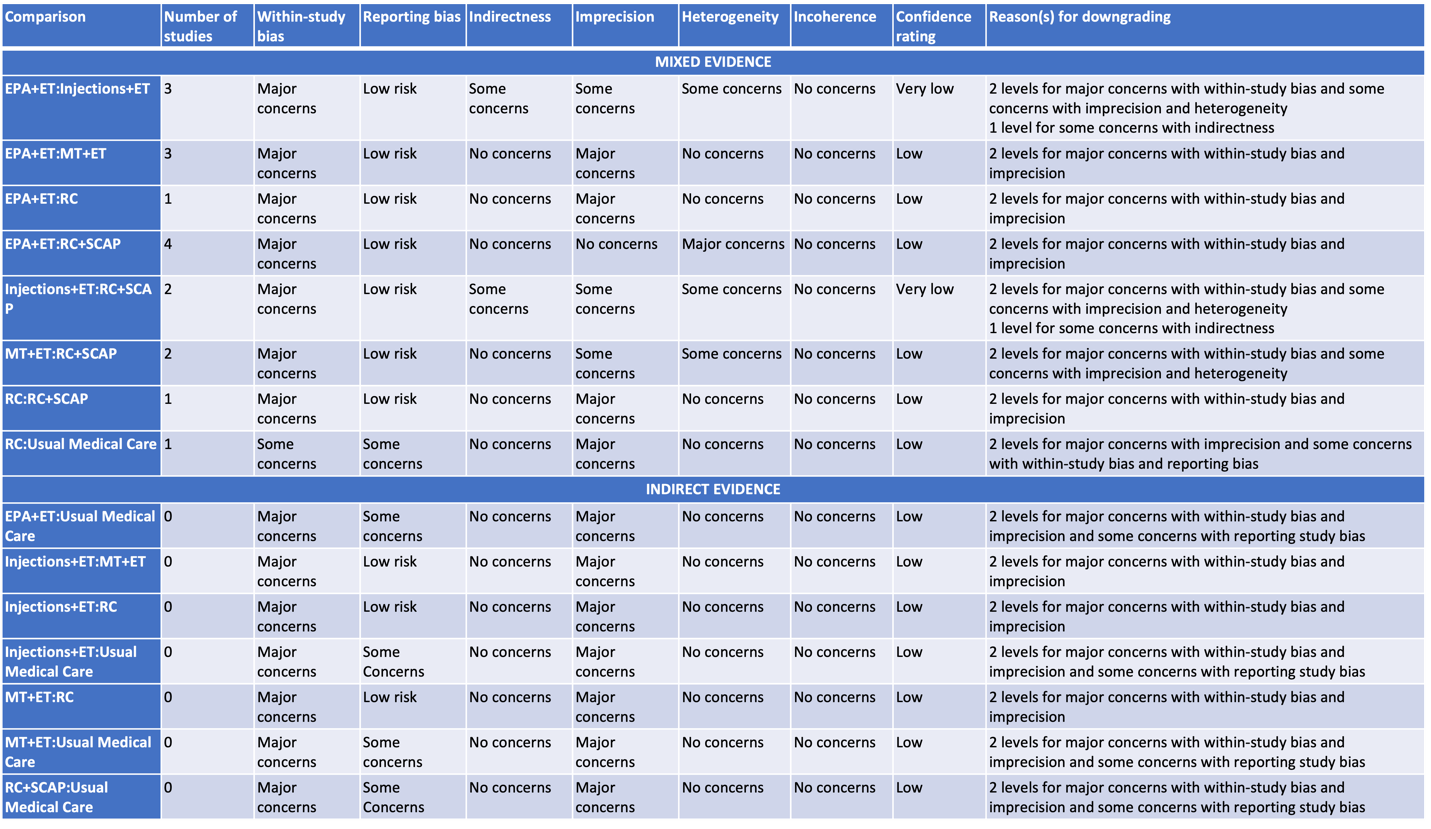


# ROM_ABD


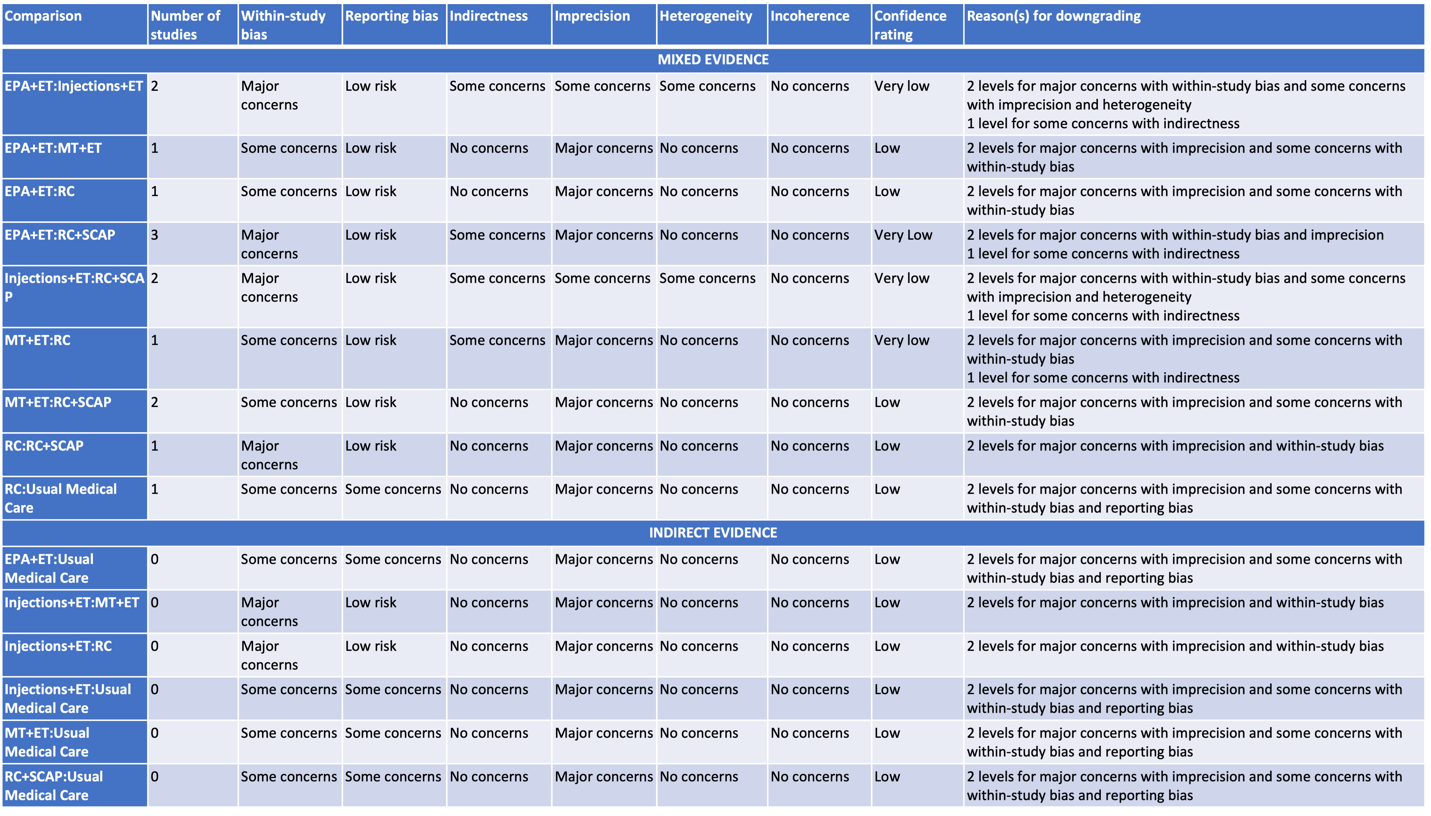


# SPADI


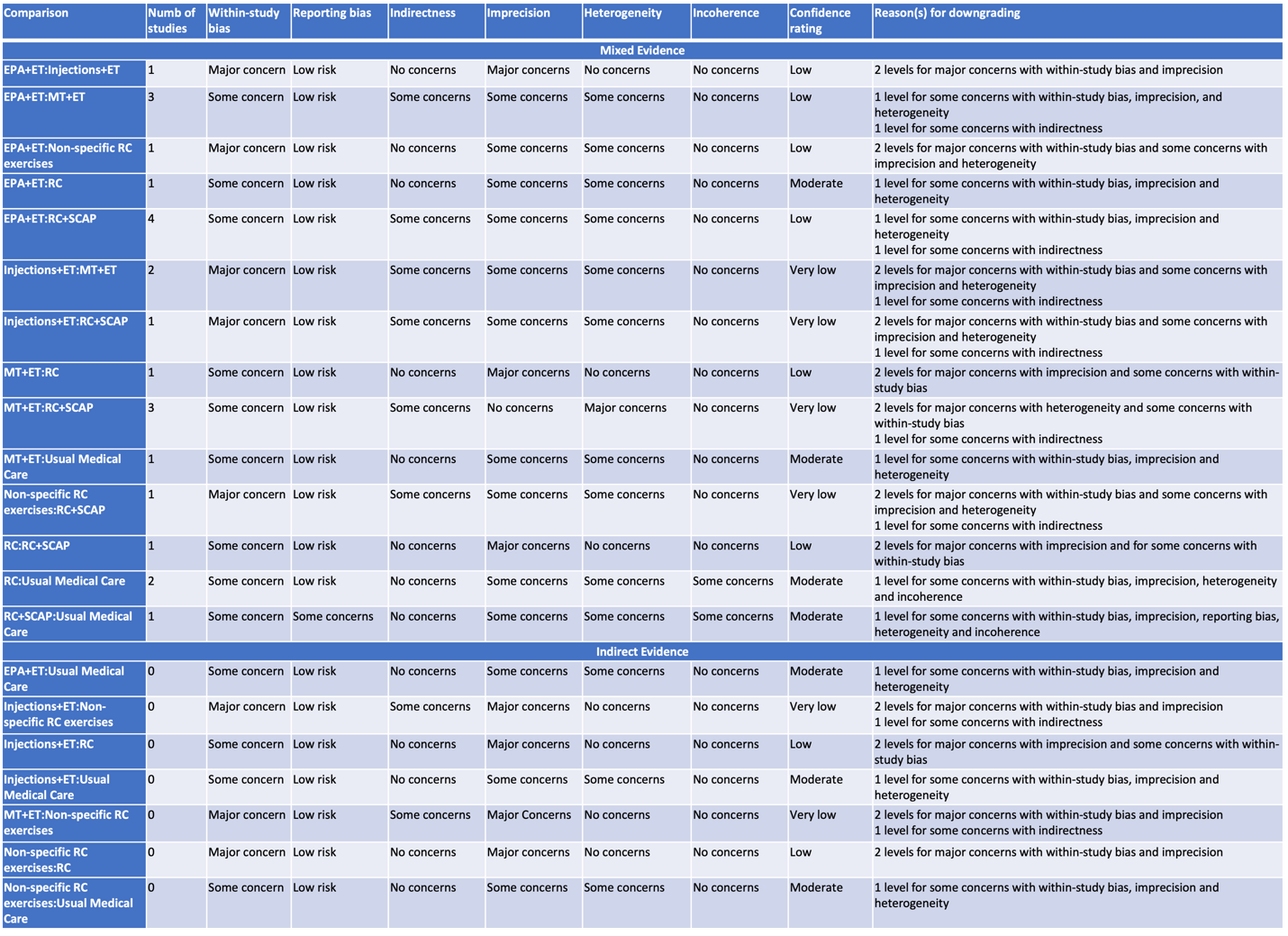


# DASH


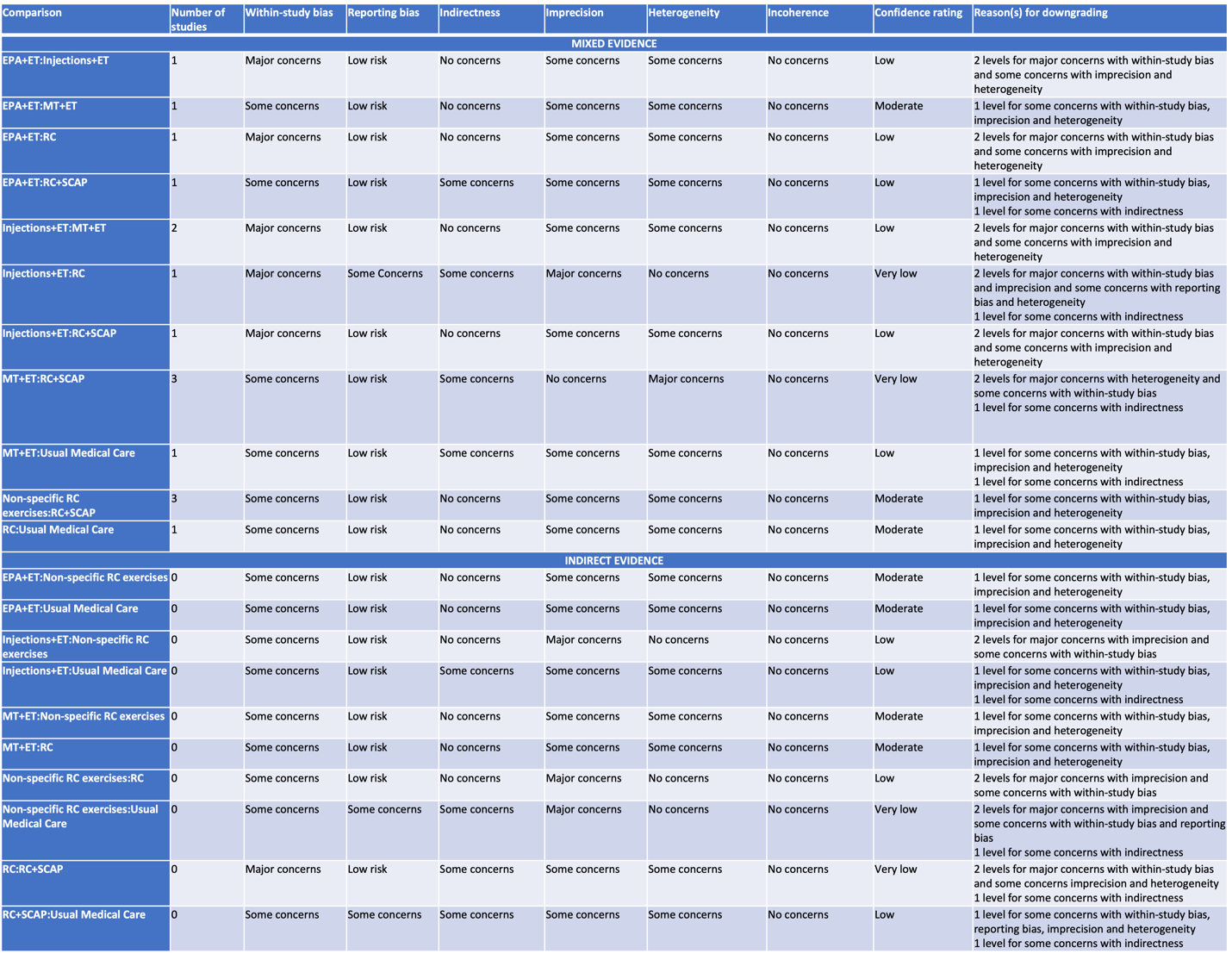

Supplement: S7 Appendix — (DOCX) [file pone.0294014.s007.docx]
