# Supplementary material for: Shoulder specific exercise therapy is effective in reducing chronic shoulder pain: A network meta-analysis
Source: PLoS One. 2024 Apr 29;19(4):e0294014. doi: 10.1371/journal.pone.0294014 (PMC11057978; doi:10.1371/journal.pone.0294014)

**RISK OF BIAS**

**Legend:**

Low risk

Some concerns

High risk

D1

Randomisation process

D2

Deviations from the intended

interventions

D3

Missing outcome data

D4

Measurement of the

outcome

D5

Selection of the reported result

**+**

**!**

**-**


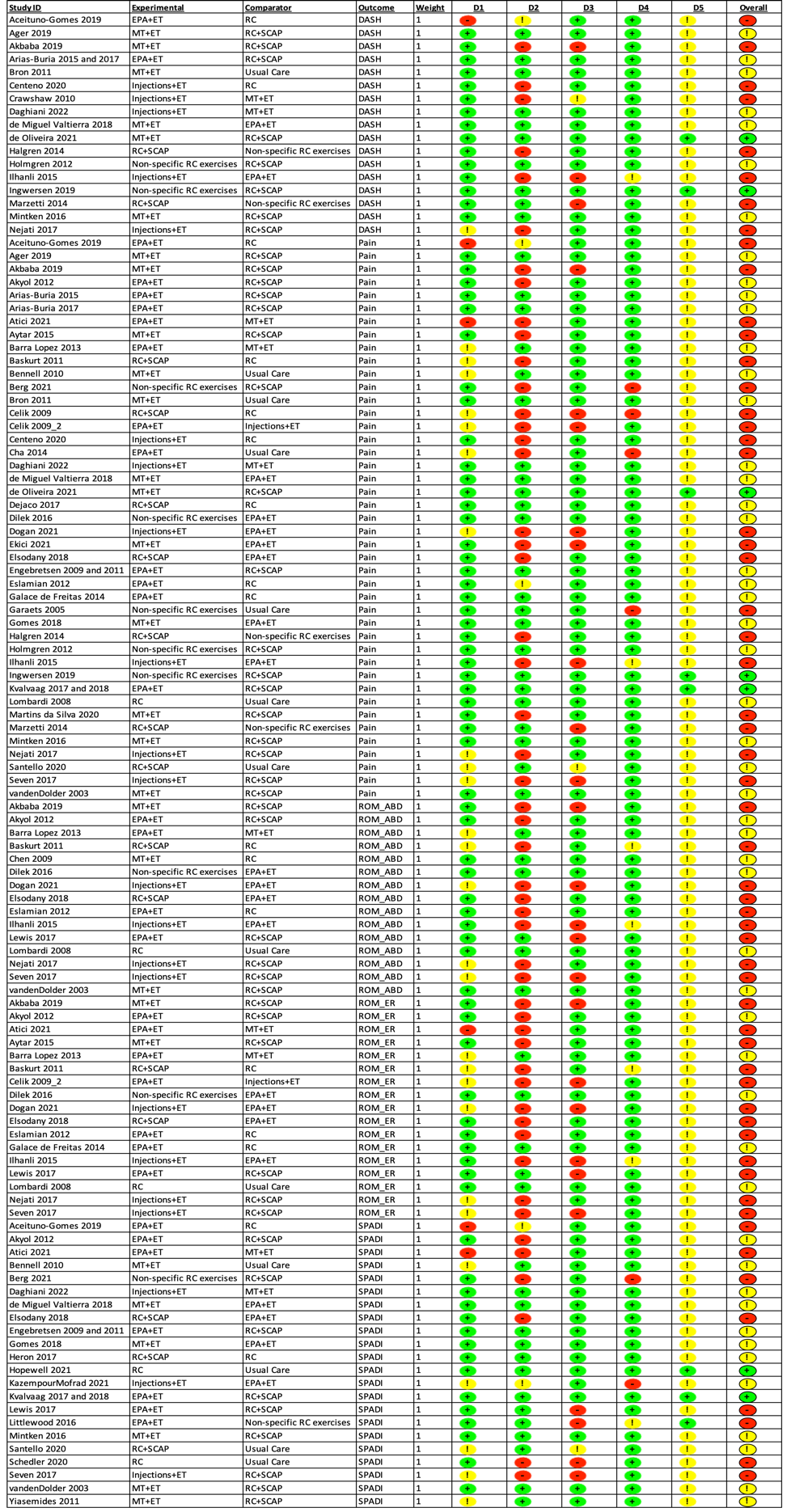

Supplement: S4 Appendix — (DOCX) [file pone.0294014.s004.docx]
