# Supplementary material for: Shoulder specific exercise therapy is effective in reducing chronic shoulder pain: A network meta-analysis
Source: PLoS One. 2024 Apr 29;19(4):e0294014. doi: 10.1371/journal.pone.0294014 (PMC11057978; doi:10.1371/journal.pone.0294014)
Supplement: Attachment — Submitted filename: Response to Reviewers_PONE-D-23-11628_08SEP2023.docx [file pone.0294014.s008.docx]

September 8, 2023

**Juan Ignacio Guerrero-Henriquez, MSc**
Academic Editor
PLOS ONE

**Dear Juan Ignacio Guerrero-Henriquez, Diego Guerra-Rodriguez and PLOS ONE reviewers,**

I hope this letter finds you well. I would like to thank you all for your invaluable feedback and constructive comments on our manuscript titled " **Shoulder specific exercise therapy is effective in reducing chronic shoulder pain: A network meta-analysis**," which was submitted to PLOS ONE for review. Your thoughtful review has been instrumental in improving the quality and rigor of our research.

I am pleased to inform you that we have carefully considered each of your comments and have made the necessary revisions to address them. Below, I have summarized your major points of concern and provided our responses and actions taken:

**Reviewer #1:**

1. **“On page 6, line 94, there seems to be a limitation regarding the eligibility criteria. The exclusion of participants with scapular dyskinesis may not fully represent the population of patients with chronic shoulder pain, many of whom present with this condition, especially those prescribed scapular exercises. Could you please clarify this point?” –** Even though scapular dyskinesis may be related to chronic shoulder pain, scapular dyskinesis is also quite common in the asymptomatic population and many symptomatic patients do not necessarily present this condition. Therefore, we excluded scapular dyskinesis, since it could be a potential confounder to our findings. (1, 2)
2. **“On page 8, line 137, it's important to note that NMA includes the assumption of consistency/transitivity. This aspect should be explicitly outlined. It would be beneficial if the authors could provide additional information on the NMA approach via CINeMA and the R Net-Meta package.”** – Information added to lines 149-153.
3. **“On page 9, line 165, the description of group classification is overly lengthy. Please consider revising for conciseness and clarity.”** – Please see revised group classification (lines 177 and 179).
4. **“On page 10, lines 174 and 182, the classification of the EPA+ET group (electro-physical agents and exercise therapy) may not be entirely appropriate. Modalities such as electrotherapy, thermotherapy, and dry needling may have varying treatment effects on shoulder pain. Additionally, the combination of data for the injection+ET group may be questionable, given that steroids, prolotherapy, and PRP each have different mechanisms and effects. Would it be possible for the authors to perform a subgroup analysis or additional analysis to address these concerns?”** – We agree that the classification of groups in this NMA limits the ability to effectively assess targeted treatment effects of individual interventions that may have different mechanisms and effects if considered separately. However, given the small number of studies, a sensitivity analysis would not be statistically viable. For example, for injections as grouped right now, we have 2-3 studies directly comparing ROM, Pain, SPADI and DASH. If we re-classify, we will either not be able to perform an NMA or the robustness and reliability of results can be compromised. Moreover, included studies frequently grouped electrotherapy, thermotherapy, and dry needling as one group (i.e., Physical Therapy group), so we decided to treat these interventions as one group for consistency. For injections, we grouped all types of injections in one group considering that all injections are meant to decrease pain indifferently of the type of injection applied. As the research in this area increases and more studies are published, subset analyses will be possible. We add this limitation in the limitation session of the revised manuscript (Lines 374-379).
5. **“Lastly, could you please provide the inconsistency results of the NMA?”** – Results were added to the revised manuscript (lines 266-267, 276-277, 297-300). Full results were also incorporated to the revised supporting material (Pages 44-50).

**Reviewer #2:** No queries.

**Reviewer #3:**

1. **“Consider describing the objective of the research clearly, because there are two different objectives throughout the publication, this can be reviewed in line 5 and line 74.”** – Changes made as requested. Please see revised manuscript (Lines 75-77).
2. **“Consider mentioning the definition of: "usual medical care" in the introduction and not only in the discussion on line 307.”** - Changes made as requested. Please see revised manuscript (Lines 55-56).
3. **“Regarding the conclusion, consider answering the objective of the research by modifying the approach of the objectives mentioned above.”** - Changes made as requested. Please see revised manuscript (Lines 414 and 416).

We believe that these revisions have significantly strengthened the manuscript by clarifying the manuscript objectives, methods, results and limitations. We appreciate the time and effort you have dedicated to the peer review process, which has undoubtedly improved the quality of our work. If you have any additional comments or concerns, please do not hesitate to contact us.

Thank you once again for your invaluable feedback and for your commitment to advancing the quality of research in our field. We look forward to your positive response and the possibility of seeing our work published in PLOS ONE.

Sincerely,

Anelise Silveira, PT, MSc, PhD Candidate

REFERENCES

1.	Hickey D, Solvig V, Cavalheri V, Harrold M, McKenna L. Scapular dyskinesis increases the risk of future shoulder pain by 43% in asymptomatic athletes: a systematic review and meta-analysis. Br J Sports Med. 2018;52(2):102-10.

2.	Salamh PAH, W.J.; Boles, T.; Holmes, D.; McMillan, A.; Wagner, A.; Kolber, M.J. Is it Time to Normalize Scapular Dyskinesis? The Incidence of Scapular Dyskinesis in Those With and Without Symptoms: a Systematic Review of the Literature

. International Journal of Sports Physical Therapy. 2023;18(3):558-76.
